# AGI-134: a fully synthetic α-Gal glycolipid that converts tumors into in situ autologous vaccines, induces anti-tumor immunity and is synergistic with an anti-PD-1 antibody in mouse melanoma models

**DOI:** 10.1186/s12935-019-1059-8

**Published:** 2019-12-19

**Authors:** Stephen M. Shaw, Jenny Middleton, Kim Wigglesworth, Amber Charlemagne, Oliver Schulz, Melanie S. Glossop, Giles F. Whalen, Robert Old, Mike Westby, Chris Pickford, Rinat Tabakman, Irit Carmi-Levy, Abi Vainstein, Ella Sorani, Arik A. Zur, Sascha A. Kristian

**Affiliations:** 1 Agalimmune Ltd., Sandwich, Kent, UK; 2 BioLineRx Ltd, Modi′in-Maccabim-Re′ut, Israel; 3 Department of Surgery, University of Massachusetts Medical School, Worcester, MA USA; 4 Immunobiology Laboratory, The Francis Crick Institute, London, UK; 5 Department of Cancer Biology, University of Massachusetts Medical School, Worcester, MA USA; 6 Wolfson Institute of Preventive Medicine, Queen Mary University of London, London, UK

**Keywords:** Immunotherapy, alpha-Gal, anti-Gal, Melanoma, anti-PD-1, Checkpoint inhibition, Cancer vaccine, AGI-134, Intratumoral injection, Abscopal effect

## Abstract

**Background:**

Treatments that generate T cell-mediated immunity to a patient’s unique neoantigens are the current holy grail of cancer immunotherapy. In particular, treatments that do not require cumbersome and individualized ex vivo processing or manufacturing processes are especially sought after. Here we report that AGI-134, a glycolipid-like small molecule, can be used for coating tumor cells with the xenoantigen Galα1-3Galβ1-4GlcNAc (α-Gal) in situ leading to opsonization with pre-existing natural anti-α-Gal antibodies (in short anti-Gal), which triggers immune cascades resulting in T cell mediated anti-tumor immunity.

**Methods:**

Various immunological effects of coating tumor cells with α-Gal via AGI-134 in vitro were measured by flow cytometry: (1) opsonization with anti-Gal and complement, (2) antibody-dependent cell-mediated cytotoxicity (ADCC) by NK cells, and (3) phagocytosis and antigen cross-presentation by antigen presenting cells (APCs). A viability kit was used to test AGI-134 mediated complement dependent cytotoxicity (CDC) in cancer cells. The anti-tumoral activity of AGI-134 alone or in combination with an anti-programmed death-1 (anti-PD-1) antibody was tested in melanoma models in anti-Gal expressing galactosyltransferase knockout (α1,3GT^−/−^) mice. CDC and phagocytosis data were analyzed by one-way ANOVA, ADCC results by paired t-test, distal tumor growth by Mantel–Cox test, C5a data by Mann–Whitney test, and single tumor regression by repeated measures analysis.

**Results:**

In vitro, α-Gal labelling of tumor cells via AGI-134 incorporation into the cell membrane leads to anti-Gal binding and complement activation. Through the effects of complement and ADCC, tumor cells are lysed and tumor antigen uptake by APCs increased. Antigen associated with lysed cells is cross-presented by CD8α+ dendritic cells leading to activation of antigen-specific CD8+ T cells. In B16-F10 or JB/RH melanoma models in α1,3GT^−/−^ mice, intratumoral AGI-134 administration leads to primary tumor regression and has a robust abscopal effect, i.e., it protects from the development of distal, uninjected lesions. Combinations of AGI-134 and anti-PD-1 antibody shows a synergistic benefit in protection from secondary tumor growth.

**Conclusions:**

We have identified AGI-134 as an immunotherapeutic drug candidate, which could be an excellent combination partner for anti-PD-1 therapy, by facilitating tumor antigen processing and increasing the repertoire of tumor-specific T cells prior to anti-PD-1 treatment.

## Background

Cancer immunotherapy has revolutionized cancer treatment, with therapies that block immune checkpoints demonstrating remarkable efficacy in a wide range of tumor types [1]. However, there are still a vast number of patients who are refractory to these treatments. The patients for whom current immunotherapies are not efficacious have tumors that have little inflammation and T cell infiltration, so-called immunologically cold tumors [2]. To boost the efficacy of the immune checkpoint inhibitors in refractory patient populations, there are several approaches that specifically aim to either: (1) produce intratumoral inflammation, (2) increase tumor antigen processing and prime naïve T cells against tumor-specific antigens (TSAs) or (3) relieve immunosuppression in the tumor microenvironment [3, 4].

One method that has been explored to boost tumor tissue processing and produce activation of TSA-specific T cells harnesses the ability of pre-existing natural antibodies to mediate rejection of xenogeneic tissue bearing Galα1-3Galβ1-4GlcNAc (α-Gal) epitopes. Humans, apes and Old World monkeys do not express the α-1,3-galactosyltransferase (α1,3GT) enzyme, which catalyzes the synthesis of α-Gal epitopes presented on cell surface glycolipids and glycoproteins in other mammals [5]. Humans therefore recognize α-Gal epitopes as foreign and, through constant antigenic stimulation by commensal gut bacteria expressing α-Gal-like epitopes, produce anti-α-Gal antibodies (termed anti-Gal) in titers as high as 1% of total immunoglobulin throughout life [6–8]. When anti-Gal antibodies bind to α-Gal-bearing tissue, they activate the complement cascade and initiate antibody-dependent cellular cytotoxicity (ADCC) [9–11], resulting in the release of inflammatory mediators and lysis of the tissue. It is the activation of complement and ADCC by anti-Gal that drives the hyperacute rejection of xenotransplants in humans.

The hyperacute response driven by anti-Gal binding to α-Gal-positive tissue can also stimulate adaptive immunity to non-self-antigens within the target tissue [12–16]. Tissue lysis during hyperacute rejection produces cellular debris that is immune-complexed with anti-Gal IgG and various complement proteins, while complement activation will cause the release of inflammatory mediators such as the anaphylatoxins C3a and C5a, which create an inflammatory tumor microenvironment that is optimal for the chemotactic recruitment and activity of antigen presenting cells (APCs), such as macrophages and dendritic cells (DCs). Immune-complexed antigens are taken up by APCs via activating Fcγ (FcγR) and complement (CR) receptors and subsequently presented to T cells [17–20]. Accordingly, viral vaccines expressing α-Gal epitopes, which form in situ immune complexes with anti-Gal, are 30- to 100-fold more immunogenic than the same vaccines lacking α-Gal epitopes [21–23]. Furthermore, tumor cell vaccines engineered to express α-Gal are more immunogenic than parental tumor cells, while also providing greater protection from subsequent parental tumor cell challenge [12].

To develop an α-Gal-based immunotherapy, it was of interest to determine whether tumor lesions can be labeled with α-Gal epitopes in order to convert them into in situ vaccines that elicit a protective immune response against autologous TSAs on tumor cells specific to individual patients by harnessing the natural anti-Gal antibody. To that aim, Galili et al. developed an α-Gal glycolipid preparation that was extracted from rabbit erythrocytes and could be injected directly into cancer lesions [15, 16]. The aim of this intratumoral administration of α-Gal glycolipids was to label the tumor with α-Gal, which would initiate an immunological cascade within the tumor that would ultimately create lasting immunity to the patient’s own TSAs. When cells were treated with the α-Gal glycolipids in vitro, the lipid component spontaneously and stably is inserted into the cell plasma membranes, presenting α-Gal epitopes for complexation with anti-Gal antibodies, which resulted in complement-dependent cytotoxicity (CDC) and cell lysis after incubation in human serum [15, 16]. When the α-Gal glycolipids were administered to primary melanoma lesions in anti-Gal producing α-1,3-galactosyltransferase knockout (α1,3GT^−/−^) mice, i.e., mice lacking α-Gal epitopes, the mice were protected from the development of secondary untreated lesions [15, 16]. The anti-tumoral effect was demonstrated to be driven by the generation of melanoma-associated antigen (MAA)-specific CD8+ T cells, which protected the mice from challenge with melanoma cells [16]. In two small scale Phase I clinical trials, the intratumoral administration of these α-Gal glycolipids was demonstrated to be well tolerated and safe for use in human cancer patients [28, 29].

Due to the impracticality of developing a human therapeutic from a crude biological extract, we explored the use of a fully synthetic α-Gal glycolipid-like molecule, AGI-134, which is a small molecule with a simple, robust manufacturing route amenable to full clinical development. AGI-134 is a Function-Spacer-Lipid (FSL) molecule that was originally developed by KODE Biotech (Auckland, New Zealand) and is comprised of a lipid tail linked to the α-Gal epitope by an adipate linker [30]. AGI-134 retains the immunological properties of naturally occurring α-Gal glycolipids extracted from rabbit erythrocytes. Here we demonstrate that AGI-134 spontaneously incorporates into human and mouse tumor cells and binds anti-Gal antibodies, leading to lysis of the AGI-134-treated cells through the activation of complement and ADCC. Complement killed cells were specifically phagocytosed by human APCs and murine CD8α+ dendritic cells, which cross-presented antigen to CD8+ T cells. In mouse models of melanoma, intratumoral administration of AGI-134 evoked primary tumor remission and an abscopal effect that protected mice from the development of un-injected distant lesions. Finally, we present evidence that the anti-tumor efficacy of AGI-134 is synergistic with an anti-programmed cell death 1 receptor (PD-1) antibody, suggesting that AGI-134 may be an excellent combination partner for synergy with checkpoint inhibitor antibodies.

## Methods

### Test compounds

AGI-134 is an FSL (Function-Spacer-Lipid) molecule originally developed by KODE Biotech (Auckland, NZ). It is an amphiphilic, water dispersible construct, which consists of an α-Gal trisaccharide functional head group, a spacer, and a diacyl lipid tail. In addition to AGI-134, two other FSL molecules were used in this study: FSL-A (functional group: blood group A trisaccharide [31]) and FSL-Fluorescein (functional group: fluorescein [32]). See Additional file 1: Fig. S1 for the FSL compound structures.

### Cell lines and primary cells

SW480 human colon adenocarcinoma cells, A549 human lung carcinoma and Chinese hamster ovary (CHO-K1) cells were purchased from the European Collection of Cell Cultures (ECACC). B16-F10 mouse melanoma cells were obtained from the American Type Culture Collection. These cell lines were authenticated at the respective cell banks via Short Tandem Repeat profiling. JB/RH mouse melanoma cells were gifted to Dr. Whalen. Ovalbumin expressing CHO-K1 (CHO-OVA) cells were generated for this study by standard retroviral transduction techniques. Briefly, retroviral particles were generated by lipid-based transfection of a host cell line with packaging plasmids and a pMSCV expression vector encoding a fusion protein consisting of the non-secretable form of OVA and the red fluorescent reporter protein mCherry. Retroviral particles were concentrated by ultracentrifugation and used to transduce CHO-K1 cells. Successful transduction was confirmed by flow cytometric analysis of transduced CHO-K1 populations, showing the presence of the red fluorescent reporter protein. The green fluorescent protein (GFP)-expressing mouse tumor dendritic cell (DC) line (MutuDC) was previously described [33]. DNGR-1 knock out (DNGR-1^−/−^) MutuDCs were generated by CRISPR/Cas9 technology as described [34]. OT-I CD8^+^ T cells, whose T cell receptors recognize the H-2K^b^-restricted SIINFEKL OVA peptide, were collected as follows: the lymph nodes and spleen of one OT-I x recombination activating gene 1 (Rag1) knock out mouse were collected and homogenized to obtain a single cell suspension. Red blood cells were lysed. The residual cells were washed and cultured for 2 days in RPMI-1640 supplemented with fetal bovine serum (FBS), β-mercaptoethanol, glutamine, penicillin/streptomycin, sodium pyruvate and non-essential amino acids in 24-well tissue culture plates at 5 × 10^4^–1 × 10^5^ cells/well, in the presence of 0.1–1 nM SIINFEKL. Mouse IL-2 was added to a final concentration of 250 units/mL on day 3 and the cultures maintained for another 2 days. On day 5, effector CD8+ T cells were enriched from the cultures by magnetic activated cell sorting using a negative depletion antibody cocktail (Miltenyi Biotec).

Human peripheral blood mononuclear cells (PBMC) were prepared from individual donor leukocyte cones (obtained from the National Health Service Blood and Transplant (NHSBT), United Kingdom) by density gradient centrifugation over Ficoll-Paque Plus (GE Healthcare Lifesciences). NK cells were enriched from freshly isolated PBMC using an eBioscience human NK cell negative selection kit and cultured overnight in complete NK medium (DMEM supplemented with FBS, l-glutamine, sodium pyruvate, non-essential amino acids and penicillin/streptomycin) in the presence of 150 units/mL of recombinant human IL-2 (Peprotech) at 37 °C, 5% CO_2_. Human PBMC were differentiated into macrophages as follows: 2 h after adding PBMCs to 6-well plates, adherent cells were washed and differentiated by 6- to 7-day incubation in medium with 100 ng/mL Macrophage Colony Stimulating Factor (M-CSF; Peprotech); Fcγ and complement receptor expression was verified by flow cytometry (Additional file 2: Fig. S2).

### α-1,3-Galactosyltransferase knockout (α1,3GT^−/−^) mice, anti-Gal induction and titer measurement

Male and female α1,3GT^−/−^ animals with an age of up to 8 months and up to 35 g in weight were used in this study. The strain was generated on a C57BL/6xDBA/2Jx129sv background with H2^b^ × H2^d^ haplotypes [15, 35] and then interbred. To induce anti-Gal antibody production, the mice received repeated intraperitoneal (i.p.) immunizations with pig kidney homogenate (PKH) [15, 36]. Anti-Gal titers were determined as described [37]: 96-well plates were coated with bovine serum albumin (BSA)-conjugated to α-Gal (α-Gal-BSA; V-Labs) or control BSA. After blocking with casein buffer (Thermo Scientific), mouse plasma samples diluted in blocking buffer were added. Bound antibodies were detected with HRP-conjugated anti-mouse antibodies, HRP substrate, sulfuric acid stop solution, and measuring 492-nm absorbance.

### Polyclonal human anti-Gal IgG purification

Anti-Gal IgG was affinity purified from human serum immunoglobulin (Baxter) at Rockland Immunochemicals (Pottstown, PA). α-Gal conjugated to human serum albumin (α-Gal-HSA; V-Labs) was immobilized to UF4 or NHS-activated Sepharose 4 Fast Flow resins (GE Healthcare); bound anti-Gal was eluted by low pH, dialyzed 3× in PBS, and sterile-filtered.

### Binding of mouse and human anti-Gal antibodies to AGI-134-treated mouse and human cancer cells

5 × 10^5^ cancer cells were treated with AGI-134 or controls in phosphate buffered saline (PBS) with rotation for 1–2 h at 37 °C. After three PBS washes, FSL-Fluorescein-treated cells could be directly analyzed; AGI-134- and FSL-A-treated cells were incubated with either: α1,3GT^−/−^ mouse or human serum, chimeric anti-Gal with human Fc portion (Absolute Antibody, Oxford, UK), affinity purified human anti-Gal, or anti-blood Group A,B antibody in PBS, 0.1% BSA or RPMI-1640 and then with fluorescein isothiocyanate (FITC)-labeled secondary antibodies (Biolegend and Sigma) prior to analysis by flow cytometry or fluorescence microscopy. To visualize cell nuclei in the microscopy samples, the test samples were stained with 4′,6-diamidino-2-phenylindole (DAPI; Abcam). Fluorescence pictures were taken using a dual band filter set allowing for simultaneous visualization of DAPI and FITC fluorescence.

### Complement deposition and complement-dependent cytotoxicity (CDC) experiments

For complement deposition assays, AGI-134- or FSL-A-treated cells were incubated with 2.5–50% pooled normal human serum (NHS; Innovative Research) or α1,3GT^−/−^ mouse serum for 10–45 min at 37 °C (modified from 62). Then, the cells were washed three times with cell staining buffer (CSB, Biolegend) and then incubated with anti-C3b/C3bi (Thermo Scientific) or anti-C5b-9 membrane attack complex (MAC; Quidel) antibodies in CSB for 30 min on ice, then washed 3×, incubated with FITC- or allophycocyanin-conjugated secondary antibodies for 30 min on ice, washed again, and subjected to flow cytometry.

For CDC assays, 5 × 10^5^ human SW480 or A549 cells were treated with AGI-134 or vehicle for 2 h at 37 °C with rotation. After three PBS washes, 1 × 10^5^ cells were added to white 96-well plates in RPMI-1640 and incubated with a final concentration of 50% NHS or heat-inactivated NHS (iNHS; NHS was treated for 30 min at 56 °C to obtain iNHS). To verify MAC-contribution to complement-mediated killing, some experiments with SW480 cells were conducted with C7-depleted human serum ± physiological C7 amounts (70 μg/mL; both Quidel). The plates were incubated for 1–2 h at 37 °C, 5% CO_2_. To measure cell viability, the CellTiter-Glo Luminescent Cell Viability Assay (Promega), which measures ATP as viability indicator, was used. For NHS vs. iNHS dose response assays, the average luminescence of untreated cells was set as 100% viability. For experiments with C7-depleted serum ± C7, the mean luminescence of cells incubated with C7-depleted serum was set as 100%.

### Antibody-dependent cell-mediated cytotoxicity (ADCC) reporter assay experiments

An ADCC Reporter Bioassay Core Kit (Promega) was used according to the manufacturer’s instructions. Briefly, A549 cells were rotated in PBS ± 0.5 mg/ml AGI-134 for 1 h at 37 °C. Cells were then washed 3× in ice-cold PBS and then added to 96-well assay plates at 3 × 10^3^ viable cells/mL in kit assay buffer. Effector and target cells were incubated at a 10:1 ratio in assay buffer containing 0 or 30 µg/mL affinity purified human anti-Gal IgG. After a 6-h incubation at 37 °C, 5% CO_2_, Bio-Glo luciferase reagent was added to each well and relative light units (RLU) measured using a bioluminescence plate reader. Blank RLU values were obtained by averaging the RLU values obtained for wells containing assay buffer and Bio-Glo luciferase reagent only. Fold-ADCC induction was calculated as [RLU in the presence of anti-Gal − Blank RLU]/[RLU in the absence of anti-Gal − Blank RLU]. Within each assay run, fold-ADCC induction in the presence of AGI-134 was normalized against fold-ADCC induction in the absence of AGI-134.

### Primary NK cell-mediated ADCC cell killing experiments

CHO-K1 target cells were stained with 1.25 µM of the green fluorescent dye carboxyfluorescein succinimidyl ester (CFSE) and cultured overnight in F12-Ham medium supplemented with FBS and l-glutamine at 37 °C, 5% CO_2_. Then, the cells were harvested and rotated in the dark in PBS ± 1 mg/mL AGI-134 for 1 h at 37 °C. After three washes with ice-cold PBS, the target cells were added to a round-bottom 96-well plate at 2–3 × 10^4^ viable cells per well and incubated with or without 20–30 µg/mL affinity purified human anti-Gal IgG for 45 min at 4 °C in the dark. Human NK effector cells were isolated and incubated in IL-2 as described above. NK effector and CFSE-labeled target cells were co-incubated at a ratio of 8:1 for 4 h at 37 °C, 5% CO_2_ in the dark. Then, the viability dye 7-Aminoactinomycin D (7-AAD; Biolegend) was added to each sample and incubated for at least 5 min at 4 °C in the dark before flow cytometric analysis. CFSE-positive target cells populations were identified in forward scatter (FSC) vs. FL-1 dot blots. The percentage of dead cells in the target cell population was determined as the percentage of CFSE+ 7-AAD+ cells. Percentage ADCC was calculated as the percentage cell death in the presence of anti-Gal IgG minus the percentage cell death in the absence of anti-Gal IgG.

### Human macrophage phagocytosis experiments

Human monocyte-derived macrophages (MDMs) were prepared as described above, then intracellularly stained with 200 nM Far Red CellTrace (Life Technologies) in PBS for 20 min at 37 °C, washed, and incubated overnight in culture medium + 100 ng/mL M-CSF. A549 target cells were labeled with 1.25 μM CFSE in PBS, 0.1% BSA for 10 min, washed with PBS, and incubated overnight at 37 °C, 5% CO_2_. 2.5 × 10^6^ CFSE-labeled A549 cells/mL in RPMI-1640 were incubated with 0–500 μg/mL AGI-134 for 2 h at 37 °C with rotation. After three PBS washes, the cells were incubated for 45 min at 5 × 10^6^ cells/mL in RPMI-1640 (no serum control) or RPMI-1640, 50% NHS as anti-Gal and complement source. Subsequently, the cells were washed and quantified. 3 × 10^5^ opsonized A549 cells were then added to 1 × 10^5^ macrophages in 200 μL culture medium. The samples were incubated at 37 °C for 2 h. For analysis, the cell mixture was washed once and the macrophages stained with PE/Dazzle 594-labeled anti-CD11b antibody (Biolegend) and then washed. In experiments to demonstrate internalization of A549 cells by macrophages, 5 μM cytochalasin D (Sigma-Aldrich) was used as phagocytosis inhibitor [38] and 0.25% trypsin–EDTA (Sigma-Aldrich) was used to detach adherent A549 cells from macrophages. In these experiments, the MDMs were intracellularly stained with 200 nM Far Red CellTrace (Life Technologies) in PBS for 20 min at 37 °C, washed, and incubated overnight in culture medium + 100 ng/mL M-CSF. After co-incubation of Far Red-labeled macrophages with CFSE-labeled A549 cells, as described above, 0.25% trypsin–EDTA was used to separate macrophages that had adhered to, rather than internalized, A549 cells. All samples were analyzed by flow cytometry. CD11b-positive cells (macrophages) that were also CFSE-positive were defined as macrophages with cell-associated (adherent or phagocytosed) A549 target cells. In samples in which trypsin/EDTA was used, FarRed+ cells (macrophages) that were also CFSE+ were defined as macrophages with phagocytosed target cells. Cytochalasin D-treated samples served as controls to show inhibition of phagocytosis.

### Dendritic cell phagocytosis and in vitro cross-presentation experiments

CHO-K1 target cells were incubated for 1 h in PBS, 500 µg/mL AGI-134 at 37 °C. After washing, the cells were incubated with 50% pooled NHS at 37 °C for 1 h to induce serum-mediated killing of the AGI-134-treated cells. Cell death was confirmed by staining with the viability dye DAPI. After one PBS wash, the cells were labeled with the red fluorescent dye CellVue Claret (Sigma-Aldrich) per the manufacturer’s instructions. The degree of Claret dye incorporation for each target cell treatment group (AGI-134+ serum or serum alone) was determined by measuring the geometric mean fluorescence intensity (gMFI) for the Claret dye channel for each treatment group. These values were used to normalize the DC uptake data as detailed below. Stained target cells were co-cultured with MutuDC effector cells at a 1:1 ratio for 30–120 min at 37 °C, 5% CO_2_ and then analyzed by flow cytometry. Viable MutuDCs were identified as GFP+ DAPI-cells and the level of target cell uptake was determined as the degree of transfer of Claret signal to the MutuDC population. To account for any differences in the degree of initial Claret dye incorporation into the target cell treatment groups, Claret signal in the MutuDC populations following co-culture with target cells was normalized by calculating [MutuDC geo. MFI × (A:B)], where: A = lowest Claret gMFI of the two target cell treatment groups, B = Claret gMFI of the target cell treatment group co-cultured with the MutuDCs.

Cross-presentation experiments were conducted as described [34]. CHO-OVA target cells were treated with PBS, 500 µg/mL of AGI-134 at 37 °C for 1 h and then washed with refrigerated PBS. The cells were then incubated with 50% pooled NHS at 37 °C for 1 h to induce complement-mediated killing of the AGI-134-labeled cells. An aliquot of the target cells was stained with DAPI and the dead cells were quantified using liquid counting beads (Becton–Dickinson) by flow cytometry. Dead cells were added to 96-well U-bottom plates in duplicate, in complete medium (RPMI-1640 supplemented with FBS, beta-mercaptoethanol, l-glutamine, sodium pyruvate, non-essential amino acids, HEPES, and penicillin/streptomycin), in a 3-fold dilution series. Wild-type and DNGR-1 knock out (KO) MutuDC were harvested from tissue culture dishes, counted and resuspended in complete RPMI medium. MutuDCs were added to the target cells in the 96-well assay plates at varying concentrations, giving a final dead cell:MutuDC ratios of 3:1 to 1:9. MutuDC and dead target cells were co-cultured for 4 h at 37 °C, 5% CO_2_. For the soluble OVA protein control, MutuDC were co-cultured with soluble OVA protein instead of dead cells. After 4 h incubation, pre-activated OT-I CD8^+^ T cells, prepared as described above, were added to each well in complete RPMI medium at an OT-1:MutuDC ratio of 3:1 and incubated overnight at 37 °C, 5% CO_2_. IFN-γ concentrations in sample supernatants were determined by ELISA.

### Mouse melanoma models and measurement of complement activation in B16-F10 tumors

For single tumor regression experiments, the right flank of α1,3GT^−/−^ mice was shaved and 2.5 × 10^5^ B16-F10 cells administered into the flank by subcutaneous (s.c.) injection on Day 0. When the tumors reached ~ 2–4 mm in diameter they were treated twice with a 1.25 mg dose of AGI-134 delivered intratumorally (i.t.) in 50 µl of PBS, each dose administered 24 h apart. Control mice were treated intratumorally with 2 × 50 µl of PBS alone. After treatment, the tumor volume was monitored for the duration of the study. In abscopal effect studies, both flanks of α1,3GT^−/−^ mice were shaved and, on Day 0, 1 × 10^6^ B16-F10 cells or 5 × 10^5^ JB/RH cells in PBS were s.c. injected into the right flank, and 1 × 10^4^ B16-F10 cells or 2 × 10^4^ JB/RH cells injected s.c. into the contralateral flank. The resulting tumors were designated as primary (1°) and secondary (2°) tumors, respectively. When the 1° tumors reached ~ 5 mm in diameter (Day 4–5 post-grafting), they were treated with a single i.t. dose of AGI-134 in 100 µL PBS, or mock treated with PBS only. In combination experiments with anti-PD-1, 1° tumors were treated i.t. with PBS or AGI-134 as above. On Days 5, 8, or 10, the mice received the first intraperitoneal (i.p.) dose of 250 μg anti-PD-1 antibody RMP1-14 (BioXcell; [39]) in 200 µL PBS. The anti-PD-1 treatment was repeated ×3 in 3–4-day intervals. Tumor sizes were determined with calipers or Image J software [40]; tumor volumes were calculated by the modified ellipsoidal formula: Tumor volume (mm^3^) = Length [mm] × Width [mm] × Width [mm] × 0.5 [41, 42]. Mice with tumors exceeding 20 mm in diameter were euthanized according to Institutional Animal Care and Use Committee (IACUC) guidelines. To enable observation periods for up to 90 days without having to euthanize mice, 1° tumors with 10–15 mm diameter were ablated by i.t. treatment with 150 μL absolute ethanol (Sigma-Aldrich). The presence or absence of visible and/or palpable 2° tumors was assessed 2–3 times/week.

To examine complement activation after intratumoral injection of AGI-134 or PBS, B16-F10 tumors were induced on the right flanks of α1,3GT^−/−^ mice using 1 × 10^6^ cells. Between Day 5–11, the tumors were treated once i.t. with 1 mg AGI-134 in 100 μL PBS or PBS alone. After 2–2.5 h, the tumors were excised and placed in PBS with protease inhibitor cocktail to prevent further complement activation or degradation. C5a was measured in tumor homogenate supernatants by ELISA (Abcam).

### Histology

B16-F10 tumors were induced on the right flank and left flanks of α1,3GT^−/−^ mice by s.c. injecting 1 × 10^6^ melanoma cells. On Day 6, the tumors on the right flanks were treated with 100 µg FSL-Fluorescein in 100 μL PBS. As a control, the tumors on the left flanks were mock treated with 100 µL PBS. The following day, the tumors were excised and frozen in OCT compound (Tissue Tek). Tumor sections were labeled with DAPI and microscope images in the GFP (to visualize FSL-Fluorescein) and DAPI (for DNA of cell nuclei) channels were taken and electronically overlaid.

### Statistical analyses

CDC and phagocytosis data were compared by one-way ANOVA. ADCC experiments were analyzed by paired t-test. Single tumor regression studies were analyzed by repeated measures analysis and back transformed least square geometric means were calculated for fold-change analysis (SAS JMP Pro 13). Secondary tumor and ethanol treatment data were analyzed using the Mantel-Cox test. C5a complement ELISA data were assessed by Mann–Whitney test. For all statistical tests, *p* values < 0.05 were considered statistically significant.

## Results

### Human anti-Gal antibodies bind to AGI-134-treated human cancer cells

**Fig. 1:**
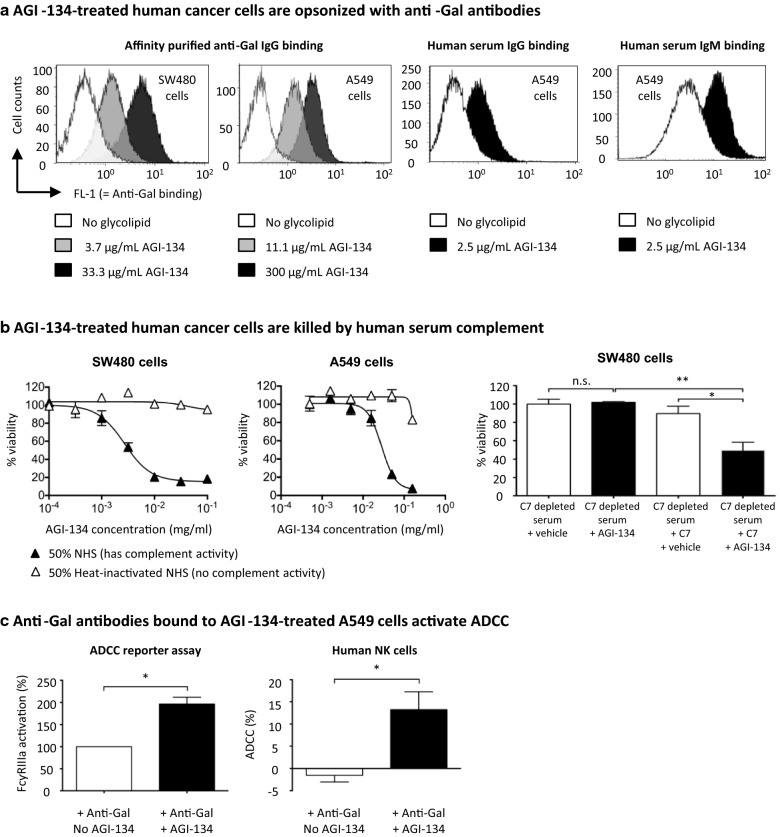
Anti-Gal binds to AGI-134-treated human cancer cells and activates CDC and ADCC. **a** Human SW480 and A549 cancer cells were treated with PBS (open histograms) or the indicated concentrations of AGI-134 (grey and black histograms). The cells were then incubated with affinity purified human anti-Gal IgG or 25% heat-inactivated human serum. Anti-Gal antibody binding was detected with fluorescently-labeled secondary antibodies and samples analyzed by flow cytometry. Representative histogram overlays from two to three independently conducted experiments for each data set are shown. **b** SW480 and A549 cells were treated with half-log dilutions of AGI-134 and incubated with 50% normal (NHS) or heat-inactivated (iNHS) human serum. In some experiments, SW480 cells were exposed to C7 depleted serum ± 70 µg/mL C7. Cell viability was determined using a luminescence-based cell viability assay and data normalized and expressed as percentage viability. Representative data from 3 independent experiments are shown, with mean values ± SD. **c** A549 cells were treated with PBS or 0.5 mg/mL AGI-134 and then co-cultured with Promega’s ADCC reporter bioassay effector cells in a 25:1 effector:target cell ratio, in the presence or absence of 30 µg/mL affinity purified human anti-Gal IgG for 6 h. Induction of ADCC over no anti-Gal antibody controls was determined by addition of Bio-Glo Luciferase reagent to quantify reporter gene expression downstream of FcγRIIIa. For assessment of target cell killing by NK cells, CHO-K1 cells were treated with PBS or 1 mg/mL AGI-134 and pre-incubated with 30 µg/mL affinity purified human anti-Gal IgG, before co-culture with IL-2-activated human NK cells. After 4–6 h of co-culture the percentage of dead CHO-K1 cells was determined by incorporation of the viability dye 7-AAD into the target cells. Data shown is the mean + SEM for three (reporter bioassay) or six (cell killing assay) independent experiments

To initiate the immunological cascade that ultimately results in antitumor immunity, AGI-134 must first incorporate into tumor cell plasma membranes and present the α-Gal antigen for binding by anti-Gal antibodies. When SW480 and A549 human cancer cells were treated with different concentrations of AGI-134, there was a concentration-dependent increase in the binding of affinity purified human anti-Gal IgG antibodies to the cells, as determined by flow cytometry (Fig. 1a). Anti-Gal in human serum also bound to AGI-134-treated cells, as when AGI-134-treated A549 cells were incubated with heat-inactivated normal human serum (iNHS), binding of IgG and IgM antibodies was observed (Fig. 1a). Furthermore, immunofluorescence experiments demonstrated that AGI-134 and anti-Gal interact on the surface of A549 cells, which is consistent with incorporation of AGI-134 into the cancer cell membranes (Additional file 3: Fig. S3A).

### Anti-Gal binding to AGI-134-treated cells activates complement and antibody-dependent cellular cytotoxicity (ADCC)

Having demonstrated that AGI-134-treated cells are opsonized by anti-Gal IgG and IgM, we next explored the effector functions elicited by these antibodies. IgM antibodies are potent activators of the classical complement pathway, while IgG antibodies can activate an array of effector functions that include complement deposition and FcγRIIIa-dependent ADCC by NK cells.

To investigate if AGI-134-mediated anti-Gal binding results in activation of complement, A549 cells were treated with AGI-134, then incubated in normal human serum (NHS) as complement and anti-Gal source before complement deposition was analyzed by flow cytometry. As anticipated, AGI-134 induced the deposition of complement C3b/C3bi and led to the formation of the membrane attack complex (MAC) C5b-C9 on A549 cancer cells (Additional file 3: Fig. S3B). Consistent with the deposition of MAC molecules, AGI-134-treated SW480 and A549 cells were killed by NHS in an AGI-134 concentration-dependent manner (Fig. 1b). The killing of the SW480 cancer cells was complement dependent, since the cells were not killed by human serum that was depleted of complement activity through heat-inactivation or removal of C7, a critical component of the MAC (Fig. 1b). When the C7-depleted serum was supplemented with a physiological concentration of human C7 (70 μg/ml), serum killing activity in the presence of AGI-134 was restored (Fig. 1b). Interestingly, the latter cell line was more resistant to CDC which may be due to higher expression of complement regulatory proteins such as CD55 and CD59 (Additional file 3: Fig. S3D). Another indicator of complement activation is the generation of the chemotactic anaphylatoxin C5a. When the assay supernatants were assayed for the presence of C5a, significantly increased C5a concentrations were observed in samples treated with AGI-134 and NHS compared to samples treated with AGI-134 and iNHS or NHS or iNHS only (data not shown).

ADCC was assessed using two separate methods: an ADCC reporter assay that measured IgG-induced FcγRIIIa activation on an ADCC reporter cell line and a second assay measuring primary human NK cell-mediated ADCC. When AGI-134-treated A549 cells were incubated with affinity purified human anti-Gal IgG and co-cultured with ADCC reporter assay effector cells, there was a two-fold increase in the amount of FcγRIIIa activation in AGI-134-treated samples compared to control samples that were treated with anti-Gal only (Fig. 1c; left graph). In experiments performed using primary blood NK cells enriched from several different donors (NK cells from a different donor were used in each independent experiment), AGI-134 treatment reproducibly induced NK-cell mediated ADCC of CHO-K1 cells (Fig. 1c; right graph).

### AGI-134-treated cells are phagocytosed by antigen presenting cells (APCs)

To initiate adaptive antitumor immune responses, the cancer cells and cellular debris created by AGI-134-induced CDC and ADCC, which is complexed with anti-Gal and complement, must be internalized and processed by APCs before TSAs can be presented to T cells.

**Fig. 2:**
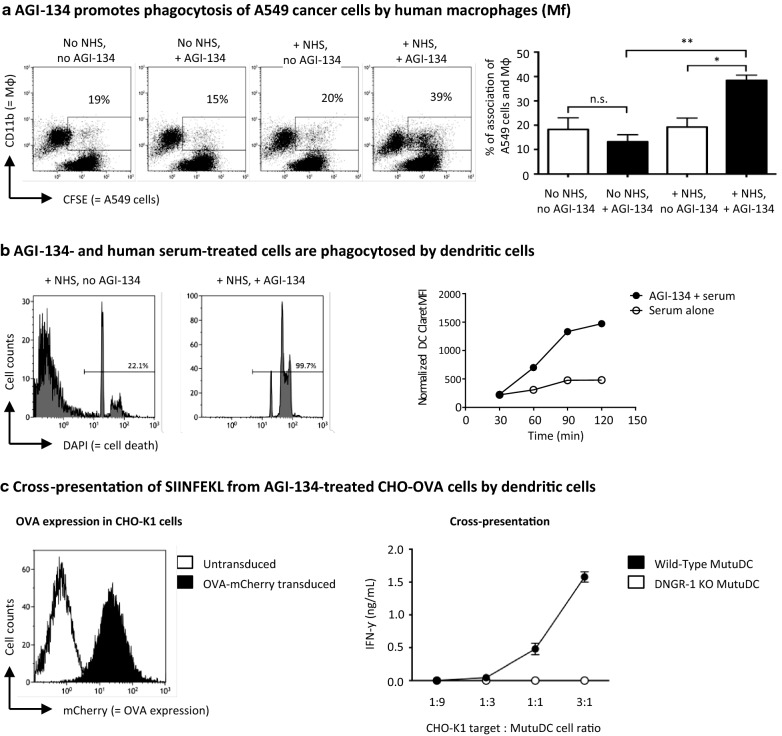
AGI-134-treated cells are phagocytosed by antigen-presenting cells and antigen cross-presented. **a** CFSE-labeled A549 cells were treated with PBS or 500 μg/mL AGI-134 and then incubated with or without normal human serum (NHS) to opsonize them with anti-Gal and complement. Subsequently, human macrophages were added at a A549 to macrophage ratio of 3:1. Subsequently, the co-cultures were stained with an anti-CD11 antibody and analyzed by flow cytometry. CFSE (for A549 cells) vs. CD11b (for macrophages) dot plots are shown for the various conditions. Double-positive events were assumed to be macrophages with associated (adherent or phagocytosed) A549 cells. In the bar graphs, the results of three independent experiments, specifically the average percentages of double positive events + SD are shown (*p < 0.05; **p < 0.005; ns, not significant; one-way ANOVA). **b** CHO-K1 cells were treated with 1 mg/ml AGI-134 and then with or without 50% NHS. Cell killing was determined by DAPI-staining of a cell aliquot. The range gates in the histogram plots quantified dead cells. The remaining CHO-K1 cells were stained with CellVue Claret dye and incubated with GFP-expressing MutuDC cells at 1:1 ratios. Samples were removed from the co-culture after 30–120 min, and analyzed by flow cytometry. The CellVue Claret dye geometric mean fluorescence intensities (gMFIs) were normalized as described in the methods and then plotted against time. **c** CHO-K1 cells were transduced to express OVA tagged with the fluorophore mCherry. The histogram shows an overlay for the mCherry signal for CHO-K1 parental cells (open curve) and CHO-OVA cells (closed curve). After treatment with vehicle or 1 mg/ml AGI-134, the CHO-OVA cells were incubated with 50% NHS before co-culture with wild-type or DNGR-1 KO MutuDCs at the indicated range of dead CHO-OVA:MutuDC cell ratios. After 4 h, OT-1 CD8+ T cells were added to the co-culture and incubated overnight. OT-1 T cell activation was quantified by IFN-γ ELISA of the co-culture supernatants

First, we studied the ability of human monocyte-derived macrophages (MDMs) to phagocytose human cancer cells that had been treated with AGI-134 and NHS. In these experiments, the A549 cells were treated with a concentration of AGI-134 (500 μg/ml) that did not induce cell killing by NHS, with the consequence that the AGI-134-treated A549 cells were viable, but opsonized with anti-Gal antibodies and complement (data not shown). When AGI-134- and NHS-treated cells were co-cultured with human MDMs, there was an approximately two-fold increase in the number of phagocytic events when compared to controls, as determined by flow cytometry (Fig. 2a). Control experiments were performed using trypsin/EDTA and the endocytosis inhibitor cytochalasin D to demonstrate that the data obtained was due to phagocytosis, and not cell–cell interactions, i.e., adherence of target to effector cells (Additional file 3: Fig. S3C).

To activate naïve antigen-specific CD8+ T cells, dendritic cells (DCs) cross-present MHC I-restricted antigen. In particular, the CD141+/XCR1+ subset in humans, and their murine CD8α+/XCR1+ counterparts, are key subsets of DCs involved in cross-presentation [43]. We therefore tested whether, like human MDMs, murine CD8α+ DCs (MutuDCs) are able to specifically phagocytose AGI-134- and NHS-treated cells. Since CHO-K1 cells are almost 100% killed by human serum after AGI-134 treatment, they were selected as the target cells. First, CHO-K1 cells were treated ± AGI-134 (1 mg/ml), incubated with NHS and cell viability determined by flow cytometry (Fig. 2b). Cells treated with both AGI-134 and NHS were almost 100% killed by human serum, whereas those treated with NHS alone were > 75% viable. After NHS treatment, the cells were loaded with the red fluorescent dye CellVue Claret and co-cultured with GFP+ MutuDCs. Phagocytosis was measured as transfer of Claret signal to the MutuDC cell population over time (Fig. 2b). There was a time-dependent increase in the transfer of Claret signal to the MutuDCs from cells that had been killed through treatment with AGI-134 and NHS, but not those live cells that had been treated with serum alone, suggesting that the killed target cells were phagocytosed by the DCs.

### Antigen from AGI-134-treated cells is cross-presented by CD8α+ DCs

Having demonstrated that MutuDCs are capable of phagocytosing CHO-K1 cells killed by AGI-134 and NHS treatment, we assessed whether antigen associated with the dead CHO-K1 cells is cross-presented. We first transduced CHO-K1 cells to express the model neoantigen ovalbumin (OVA) conjugated to the fluorescence marker mCherry (Fig. 2c). The mCherry-OVA CHO-K1 cells were treated with AGI-134 and NHS to induce CDC; the dead cells were quantified and co-incubated with wild-type or DNGR-1^−/−^ MutuDCs at dead CHO-K1:MutuDC ratios ranging from 3:1 to 1:9. After incubation, purified OT-1 CD8+ T cells, with transgenic T cell receptors that specifically recognize SIINFEKL (the immunodominant antigen of OVA) [44], were added to the co-culture. After overnight incubation, OT-1 cell activation was measured by IFN-γ ELISA. We observed that OT-1 cell activation was directly proportional to the dead CHO-K1 to MutuDC ratio (Fig. 2c). When the experiment was performed in parallel using DNGR-1^−/−^ KO MutuDCs, there was no OT-1 T cell activation. DNGR-1 is a danger associated molecular pattern (DAMP) sensing receptor on DCs that recognizes the DAMP F-actin, an event that has been demonstrated to be critical for DCs to sense dead cells [45]. DNGR-1^−/−^ MutuDCs are still able to cross-present soluble antigen, as incubation of soluble OVA with MutuDCs prior to co-culture with OT-1 cells resulted in OT-1 cell activation that was equal to wild-type cells (data not shown).

### AGI-134 binds serum anti-Gal antibodies and activates complement on treated murine cells

After demonstrating that AGI-134 treatment of cells in vitro initiates an immunological cascade that ultimately results in the activation of CD8+ T cells against cell-associated antigens, we evaluated the antitumor efficacy of AGI-134 in abscopal models of murine melanoma.

Importantly, wild-type mice and most mouse cell lines express α-Gal epitopes and thus cannot be used to test α-Gal-based immunotherapies. In contrast, α1,3GT^−/−^ mice [15, 35], like humans, do not possess a functional α1,3GT gene and therefore do not express α-Gal epitopes. Consequently, α1,3GT^−/−^ mice produce anti-Gal IgM and IgG antibodies in titers similar to those seen in humans in response to immunization with α-Gal-positive tissue [14, 35, 36, 46]. AGI-134 activity was tested in B16-F10 and JB/RH melanoma models in the α1,3GT^−/−^ mice, since these cell lines have been demonstrated to be among the few mouse cancer cells lines that do not express α-Gal [47].

We first established, as with human cancer cells, that AGI-134 incorporates into the plasma membranes of mouse B16-F10 and JB/RH cells by demonstrating the binding of monoclonal mouse anti-Gal IgM antibody to AGI-134-treated cells in vitro (Additional file 4: Fig. S4A). Next, binding of IgG and IgM antibodies from anti-Gal expressing α1,3GT^−/−^ mouse plasma to AGI-134-treated B16-F10 cells was demonstrated. Specifically, the cells were treated with or without AGI-134 (500 μg/ml) and then incubated with plasma from PKH-immunized (highly anti-Gal-positive) or, as a control, non-immunized (low anti-Gal titers) α1,3GT^−/−^ mice (see Additional file 5: Fig. S5A for representative anti-Gal titers in non-immunized and PKH-treated α1,3GT^−/−^ mice). Plasma anti-Gal binding to the treated cells was detected with a secondary antibody against both mouse IgG and IgM and the cells analyzed by flow cytometry. There was a strong increase in binding of plasma antibodies to B16-F10 cells treated with AGI-134 and incubated with anti-Gal-positive plasma, compared to untreated cells or those incubated with anti-Gal-negative plasma (Additional file 4: Fig. S4B). This data indicates that AGI-134 selectively binds anti-Gal antibodies from α1,3GT^−/−^ mouse plasma to the B16-F10 cells.

To demonstrate a functional consequence of anti-Gal binding to AGI-134 treated melanoma cells in this murine system, we incubated AGI-134-treated B16-F10 cells with anti-Gal-positive or negative α1,3GT^−/−^ mouse serum and examined complement deposition by flow cytometry. While C3b and MAC complement proteins were deposited on the cells from anti-Gal positive mouse serum, considerably less was deposited from anti-Gal negative serum (Additional file 4: Fig. S4C). Furthermore, when cells were treated with FSL-A, an analogue of AGI-134 in which the functional α-Gal group is replaced with blood group A antigen, no deposition of complement from either anti-Gal-positive or negative mouse serum was observed, further demonstrating the specificity of AGI-134 in mediating binding of anti-Gal antibodies to B16-F10 cells (Additional file 4: Fig. S4D).

### Intratumoral administration of AGI-134 into primary lesions causes tumor regression and protects mice from the development of secondary lesions

Having demonstrated that AGI-134 has a functional effect in murine in vitro systems as well as human, we next tested the efficacy of AGI-134 in models of murine melanoma in anti-Gal expressing α1,3GT^−/−^ mice.

**Fig. 3:**
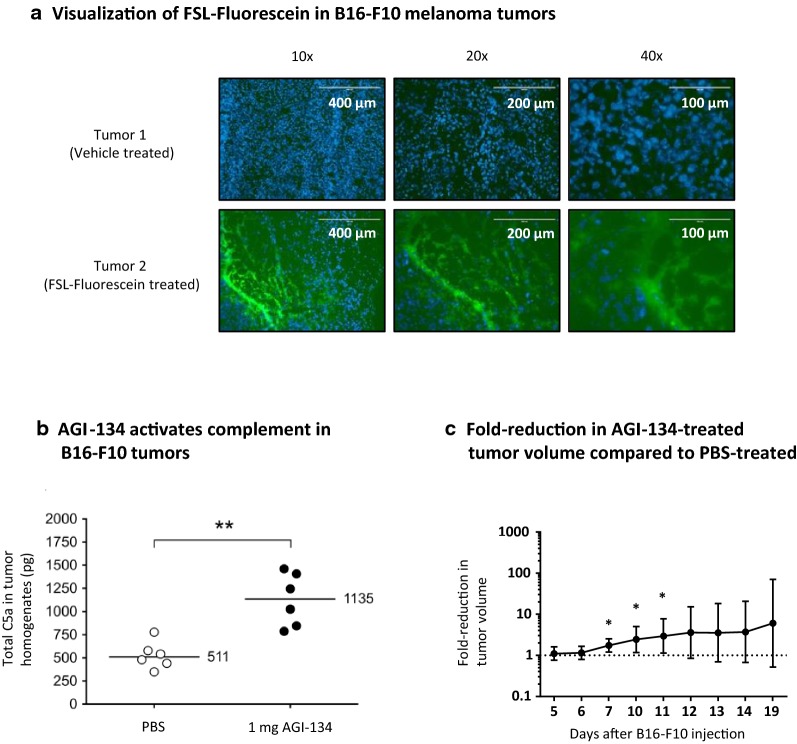
Primary tumor treatment with AGI-134 causes tumor regression, activates complement and FSL distribution in tumors. **a** Glycolipid detection in B16-F10 tumors: FSL-Fluorescein was used as surrogate molecule for AGI-134 visualization in tumors. 1 × 10^6^ B16-F10 cells were grafted onto immunized α1,3GT^−/−^ mice on both flanks. Five days later, the two tumors on each mouse were treated with 100 μL of 1 mg/mL FSL-fluorescein on one flank and with 100 μL PBS on the other flank. The following day, the tumors were excised and frozen in OCT compound. The tumors were sectioned and labeled with DAPI. Pictures in the GFP and DAPI channels for FSL and tumor cell nucleus DNA visualization were taken using ×4–×40 objectives (×10 example pictures are shown). The pictures show representative data of DAPI and GFP channel picture overlays for a vehicle- and a fluorescein-lipid treated tumor from the same mouse. **b** In complement activation experiments, B16-F10 tumors were treated by intratumoral injection of vehicle (PBS) or 1 mg AGI-134 on Day 6 post B16-F10 cell grafting. 2.5 h after treatment, tumors were excised, homogenized and the complement factor C5a measured by ELISA. Each symbol represents the total C5a in the tumor homogenate of each mouse, median C5a values are indicated by the bars. Differences between the PBS vs. AGI-134 treatment groups were assessed by Mann–Whitney test (**p < 0.003). **c** In primary tumor regression experiments, back transformed least square geometric means for PBS and AGI-134 treatments across the timepoints were calculated and fold-reduction in geometric means ± 95% CI plotted, (*p < 0.05, n = 13)

First, to examine the tumor distribution of AGI-134 after intratumoral administration, an analogue of AGI-134 in which the functional α-Gal group was replaced with fluorescein (FSL-Fluorescein) was used to allow microscopic analysis. When primary tumors were injected with FSL-Fluorescein and resected 24 h later, strong fluorescence staining of the tumor sections was observed, indicating that the glycolipid had distributed throughout the tumor and was still present 24 h later (Fig. 3a).

Having demonstrated that AGI-134 induces complement activation in vitro, we next determined if intratumoral administration of AGI-134 activated complement within injected B16-F10 tumors. Consistent with the in vitro findings, AGI-134 evoked intratumoral complement activation that resulted in significantly raised levels of C5a compared to tumors treated with vehicle alone (Fig. 3b).

To then test the ability of AGI-134 to induce regression of established tumors, a subcutaneous single flank B16-F10 tumor model in α1,3GT^−/−^ mice was used. Once the lesion had reached an injectable size (~ 2–4 mm in diameter) it was treated intratumorally with either AGI-134 or PBS and the tumor volume monitored for up to 32 days post B16-F10 cell grafting. Two intratumoral 1.25-mg doses of AGI-134, delivered 24 h apart, resulted in significant regression of the tumor when compared to mice treated with PBS alone (Fig. 3c).

**Fig. 4:**
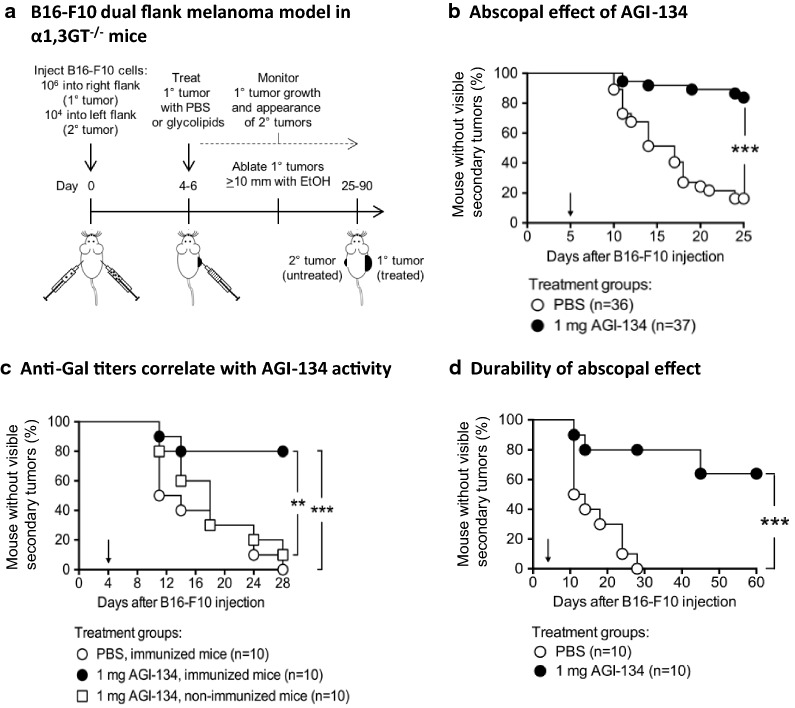
AGI-134 treatment of primary tumors produces an abscopal effect protecting mice from contralateral tumor development. **a** Schematic of abscopal B16-F10 melanoma model in anti-Gal-expressing α1,3GT^−/−^ mice. To monitor the abscopal effect of AGI-134, primary B16-F10 lesions were treated once by intratumoral injection of PBS or 1 mg AGI-134 and the development of contralateral lesions monitored. The percentages of mice without visible/palpable contralateral tumors are plotted in the graphs. The solid arrows indicate the day of AGI-134 or mock treatment (Day 4–6). **b** The pooled data from four independent experiments where the abscopal effect in B16-F10 tumors was monitored over 25 days are summarized. **c** B16-F10 tumors in immunized (anti-Gal positive) or non-immunized (anti-Gal negative) α1,3GT^−/−^ mice were treated i.t. with vehicle, or 1 mg AGI-134. **d** Representative data from two experiments where the abscopal effect of AGI-134 in B16-F10 tumors was monitored over 60–90 days is shown. Statistical differences between treatment groups in each plot were analyzed by Mantel–Cox test (**p < 0.005; ***p < 0.0005)

To examine the abscopal efficacy of AGI-134, α1,3GT^−/−^ mice were implanted with B16-F10 tumor cells to produce single primary and secondary lesions on each flank (see Fig. 4a for a schematic representation of the B16-F10 model). After the primary lesions had reached a diameter of ~ 5 mm, they were injected with a single dose of the test compound and growth of the contralateral tumor monitored for the duration of the study.

In mice bearing tumors on both flanks, a single injection of AGI-134 into primary tumors conferred significant protection from the development of uninjected tumors on the contralateral flank in four independent experiments (Table 1). When the data from the four experiments were summarized, contralateral tumors developed in 86% (31/36) of PBS-treated mice within the 25-day observation period, whereas they developed in only 16% (6/37) of AGI-134-treated mice (Fig. 4b). The efficacy of AGI-134 was dose-dependent, with a maximal abscopal effect observed with a 1-mg dose and reduced efficacy with doses of 0.5 and 0.1 mg, which were still significant when compared to mock-treated controls (Additional file 5: Fig. S5B).

**Table 1:** Abscopal effect of AGI-134 in in four independent experiments: incidence of distal B16-F10 tumor development after treatment of a primary B16-F10 tumor with AGI-134 or vehicle

Experiment #	Observation period (days)	Mice with 2° tumors at end of the observation period		Mantel–Cox test results
		AGI-134 group	PBS group	
1	25	2/10 (20%)	8/10 (80%)	**p = 0.0017
2	31	2/10 (20%)	8/10 (80%)	*p = 0.009
3	32	0/7 (0%)	7/7 (100%)	***p = 0.0002
4	32	2/10 (20%)	10/10 (100%)	***p = 0.0004

The abscopal effect of AGI-134 was completely dependent on the expression of anti-Gal antibodies. AGI-134 did not protect mice from distal tumor development that were non-immunized, and thus anti-Gal negative, but it did protect those that were expressing anti-Gal (Fig. 4c), demonstrating that the abscopal effect induced by AGI-134 in the B16-F10 model was dependent on the interaction of anti-Gal with the α-Gal portion of AGI-134.

To test the durability of AGI-134 efficacy, two longer-term experiments lasting 60 or 90 days were performed, which demonstrated that a single 1-mg intratumoral dose of AGI-134 protected mice from the development of contralateral tumors for > 60 (Fig. 4d) and > 90 days (data not shown).

It must be noted that grafting of 1 × 10^6^ B16-F10 cells creates a rapidly growing primary tumor. As AGI-134 is administered only once, and to enable longer observation periods studying the development of secondary tumors while not violating IACUC approval, which states that mice with tumors over 20 mm in diameter must be euthanized, the primary tumors were ablated by intratumoral injection with ethanol if they reached a diameter of 10 mm. Importantly, there was no significant difference in the timing or requirement for ethanol ablation of primary tumors in the PBS- or AGI-134-treated mice (29/36 PBS and 24/37 AGI-134 mice required ethanol ablation; *p *< 0.18; Mantel-Cox test, data not shown). As AGI-134 clearly protected mice from the development of contralateral tumors, we can conclude that ethanol ablation of the primary tumors did not influence the efficacy of AGI-134.

**Fig. 5:**
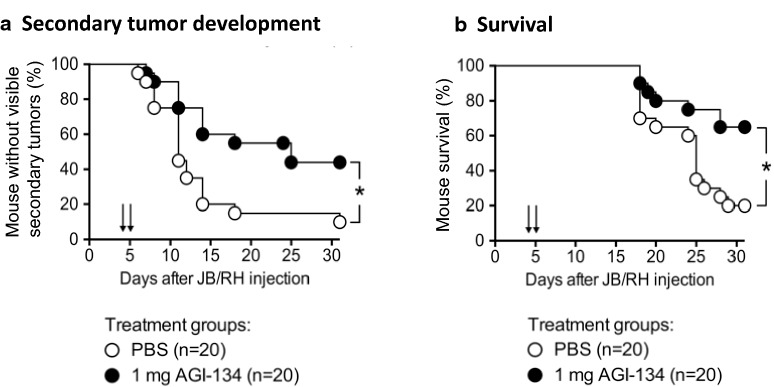
AGI-134 protects mice from secondary tumor development and improves survival in a JB/RH melanoma model. Anti-Gal-expressing α1,3GT^−/−^ mice were grafted with 5 × 10^5^ JB/RH cells to create a 1° tumor on one flank and 2 × 10^4^ JB/RH cells on the contralateral flank. 4–5 days after grafting, the 1° tumors were treated once with 1 mg AGI-134 or PBS and contralateral tumor development (**a**) and mouse survival (**b**) monitored. Pooled data from three independent experiments are shown. Statistical differences between treatment groups were analyzed by Mantel-Cox test (*p < 0.05). The solid arrows indicate the day of AGI-134 or mock treatment (Day 4 or 5)

The data on the abscopal effects of AGI-134 injected into the primary lesion were further validated in an additional model of mouse melanoma. As with B16-F10 cells, JB/RH mouse melanoma cells lack α-Gal expression [47] and thus provide an additional model in which it is feasible to investigate the anti-tumor activity of AGI-134 in α1,3GT^−/−^ mice. In the JB/RH model, a single dose of AGI-134 injected into a primary tumor significantly protected mice from the development of contralateral tumors, as well as providing a significant survival benefit (Fig. 5).

### AGI-134 boosts the antitumor efficacy of anti-PD-1 antibodies in a B16 melanoma model

Previous studies demonstrated that the abscopal anti-tumor effect conferred by intratumoral administration of rabbit erythrocyte-derived α-Gal glycolipids involved the activation of tumor-antigen specific CD8+ T cells [16]. We have demonstrated here that SIINFEKL is cross-presented to CD8+ T cells from CHO-OVA cells phagocytosed by CD8α+ murine DCs (Fig. 2c). We therefore hypothesized that the abscopal anti-tumor activity of AGI-134 in murine models of melanoma also involves the activation of CD8+ T cells and thus may be an excellent combination partner for anti-PD-1 antibodies. To test this, we performed combination studies using AGI-134 and RMP1-14, a murine specific anti-PD-1 antibody, in the α1,3GT^−/−^ mouse B16-F10 melanoma model.

**Fig. 6:**
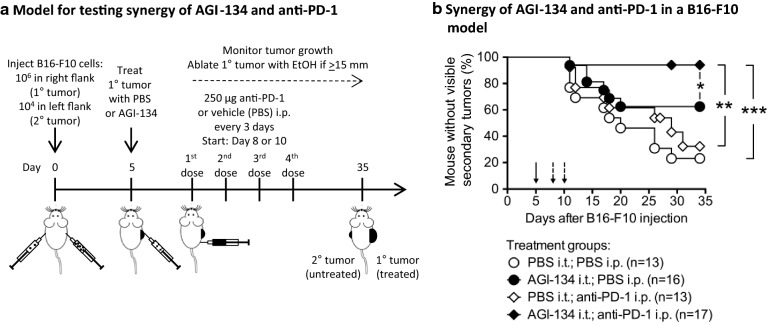
AGI-134 synergizes with an anti-PD-1 antibody. **a** Schematic for testing efficacy of AGI-134 in combination with RMP1-14, an anti-PD-1 antibody. **b** On Day 5 after B16-F10 cell grafting, mice were treated i.t. with single 100 or 250 µg doses of AGI-134 or vehicle, and then intraperitoneally with four 250-μg doses of RMP1-14 or vehicle in 3–4-day intervals starting on Day 8 (experiment #1) or Day 10 (experiment #2) post B16-F10 cell grafting. For the graph, the data from two independent experiments were combined and plotted. The data show the percentage of mice free from secondary tumors over time. The treatment groups were statistically compared by Mantel–Cox test (*p < 0.05; **p < 0.005; ***p < 0.0005). Solid arrows indicate the time of i.t. AGI-134 or vehicle treatment; dashed arrows show the start of i.p. RMP1-14 treatment

To evaluate the effects of combining AGI-134 and anti-PD-1, we first identified conditions in which each compound, when administered alone, had suboptimal efficacy in the α1,3GT^−/−^ B16-F10 mouse model. As described above, administration of 0.1-mg and 0.5-mg doses of AGI-134 to primary tumors provided protection from contralateral tumor development that was significant, but less pronounced than 1 mg AGI-134 (Additional file 5: Fig. S5B). When administered in four consecutive 0.25-mg intraperitoneal (i.p.) doses, starting at day 5 post tumor cell grafting, RMP1-14 significantly protected mice from contralateral tumor development (Additional file 5: Fig. S5C). However, when RMP1-14 treatment was started on Day 8 or 10 post tumor cell grafting, no protection was conferred (data not shown). Based upon these observations, combination experiments were performed using single doses of 0.1 or 0.25 mg of AGI-134, administered intratumorally on Day 5 post cell grafting, with four 0.25 mg i.p. doses of RMP1-14 beginning on Day 8 or Day 10 (Fig. 6a).

Of 17 α1,3GT^−/−^ mice treated with the combination of AGI-134 and RMP1-14, only one mouse (6%) developed a distal tumor within the 35-day observation period, i.e., 16 of the mice (94%) were protected (Fig. 6b). In contrast, 77% of the mock treated animals (PBS i.t.; PBS i.p.) developed a distal tumor. Importantly, the degree of protection conferred by the AGI-134/RMP1-14 combination was statistically significantly greater than that seen in the AGI-134 or RMP1-14 only groups, in which 38% and 62% of the animals developed distal lesions, respectively. Overall, these data suggest that AGI-134 has the potential to be an excellent combination partner for anti-PD-1 antibodies by activating T cells specific to the autologous TSAs prior to the expansion of the activated T cell clones by checkpoint inhibitors.

## Discussion

The natural anti-Gal antibody produces a powerful immune response that drives the hyperacute rejection of α-Gal-positive xenogeneic tissue [48, 49]. In addition to the hyperacute response, anti-Gal has been demonstrated to drive adaptive immunity to viruses that were engineered to express α-Gal epitopes [22]. These properties have led researches to assess the ability of α-Gal-based immunotherapies to treat cancer through the creation of adaptive immunity to TSAs. Two main routes have been adopted to achieve this: the first involves the in situ labelling of tumor tissue with natural α-Gal glycolipids by intratumoral injection, which aims to create immunity to each patient’s unique TSAs [15, 16]; the second involves the subcutaneous administration of allogenic whole cells that have been modified to express α-Gal, which aims to create immunity to generic TSAs [14, 24–26]. Both approaches have significant disadvantages that have been addressed in the development of AGI-134.

Since most TSAs are the result of mutations that are specific to patient, allogeneic tumor cell lines may lack many of the TSAs that could elicit a protective immune response in the individual patient [27].

Allogeneic whole cell vaccines face several issues: the cells contain abundant immunodominant antigens, such as the HLA molecules on the cells, in addition to the fact that vaccine relies upon the antigens within the delivered cells being both immunogenic and identical to those expressed by host lesions [27]. To overcome these significant issues, an α-Gal-based immunotherapy that labels a patient’s tumor mass in situ, and thereby creating immunity to the patient’s own set of unique TSAs is required. This is achieved by administering α-Gal glycolipids directly into a cancer lesion, which spontaneously inserts into the plasma membranes of the tumor cells, presenting the α-Gal epitope for binding with natural anti-Gal antibodies and driving CDC and ADCC of the tumor cells. Of note, AGI-134 may also drive localized direct killing of cancer cells as it is cytotoxic in vitro at high concentrations (data not shown). Previous pre-clinical and clinical work utilized natural α-Gal glycolipids that were extracted from rabbit erythrocytes [15, 16, 28, 29]. However, it is technically challenging to fully characterize batch-to-batch composition differences of rabbit erythrocyte-derived glycolipids, or produce them using a controlled process that enables their development as a human therapeutic. Our data provided herein suggests that the fully synthetic small molecule AGI-134 has the immunological functionality of naturally derived α-Gal glycolipids, but through having a scalable, refined and cost-effective manufacturing route, is amenable to full development as a human therapeutic.

To address how anti-Gal binding to α-Gal drives adaptive immunity to non-self antigens in the context of tumors injected with α-Gal glycolipids, the array of effector functions elicited by the polyclonal repertoire of anti-Gal antibodies must be considered. Of particular importance are the anti-Gal IgM and IgG subclasses. Anti-Gal IgM, which comprises greater than 1% of total serum IgM, is a powerful complement fixer and has been demonstrated to be largely responsible for the complement-mediated hyperacute rejection of xenogeneic transplants [50]. It is known that complement activation can induce lysis of cancer cells by CDC, through the deposition of membrane attack complex on cell surfaces. We have shown that AGI-134 mediates CDC effectively in both SW480 and A549 human cancer cells. Interestingly, the latter cell line was more resistant to CDC, i.e., more AGI-134 was necessary to facilitate A549 cell lysis by human serum which may be due to higher expression of complement regulatory proteins such as CD55 and CD59 [61]. In addition, complement activation has a number of effects that actively link innate and adaptive immunity [51, 52]. When the classical complement cascade is activated through recognition of IgM or IgG on cells, the resulting cascade results in the deposition of complement proteins [62] that can be recognized by various APCs. For example, C3b/C3bi are recognized by complement receptor 1 (CR1) on macrophages [18], while soluble antigens complexed with C3d/C3dg are bound by CR2 on follicular DCs, which present antigen to B cells during proliferation and class switching in germinal centers [53]. We have demonstrated here that C3b/C3bi is deposited on AGI-134-treated cells after incubation with human serum and that human macrophages specifically phagocytose these cells. During complement activation, the anaphylatoxins C3a and C5a are released through proteolytic cleavage of precursor proteins. In particular, C5a is a powerful chemoattractant that recruits monocytes to the site of complement activation and stimulates their differentiation into macrophages and DCs [54]. We have demonstrated that AGI-134 binds anti-Gal antibodies to treated cells, leading to deposition of complement proteins C3b/C3bi and membrane attack complex and subsequent cell lysis after incubation in human serum in vitro. Additionally, we showed that C5a was significantly more abundant in B16-F10 tumors after treatment with AGI-134 compared to tumors treated with the vehicle control, PBS. AGI-134 therefore induces the lysis of treated tumor cells, creating immune-complexed cellular debris and an inflammatory tumor microenvironment that is optimal for the uptake and processing of non-self antigens, such as TSAs, by APCs. Of note, C5a is known to be a chemoattractant for neutrophils. It would be interesting to determine in future studies if AGI-134 treatment can lead to neutrophil phagocytosis and ADCC of serum opsonized cancer cells in vitro and to neutrophil recruitment to the tumors as these effector cells may have anti-tumoral activity via ADCC and phagocytosis.

Functional anti-tumor immunity is largely driven by TSA-specific CD8+ T cells. The most important APC in activating CD8+ T cell responses are DCs, particularly the CD141+ (human) and CD8α+ (murine) conventional DC subsets, which are particularly efficient in cross-presentation of antigen to CD8+ T cells [55, 56]. DCs may ingest IgG-opsonized antigen via activating cell surface FcγRs, which promotes DC activation, maturation and translocation to secondary lymphoid tissue, where they cross-present antigen to CD8+ T cells [19, 20]. As DCs express both activating and inhibitory FcγRs, the ratio between the two receptor subtypes has an important outcome on the response of the DC to the antigen [57]. C5a, which is increased in AGI-134-treated tumors, actively promotes an increase in the ratio of activating to inhibitory FcγR on APCs, skewing the response to ingestion of IgG-tagged antigens towards activation of the DC, rather than inhibition [58]. The role of FcγR-mediated phagocytosis and processing of antigens complexed with anti-Gal was studied using influenza virus and HIV gp120 protein. In these experiments the influenza virus and HIV gp120 protein were modified to express α-Gal epitopes and then administered to anti-Gal expressing α1,3GT^−/−^ mice [21]. When compared to non-α-Gal-labeled HIV gp120, the presence of α-Gal boosted the titer of anti-gp120 antibodies by > 100-fold. Using the same animal model, immunization with α-Gal-labeled influenza virus conferred significantly higher protection from subsequent challenge with a lethal influenza dose than those immunized with non-α-Gal-labeled virus [22]. The increased protection afforded by the α-Gal labeled virus was shown to be conferred by increased virus antigen-specific CD4+ and CD8+ T cells.

We have used an in vitro cross presentation assay to demonstrate that CHO cells lysed through AGI-134-stimulated CDC and ADCC are specifically phagocytosed by murine CD8α+ DCs cells (MutuDCs). Furthermore, when OVA-expressing CHO cells were treated with AGI-134 and human serum before incubation with MutuDCs, the immunodominant peptide of OVA, SIINFEKL, was cross-presented to transgenic CD8+ T cells. Thus, AGI-134 is able to initiate an immunological cascade that results in the activation of CD8+ T cells with specificity for cell-associated antigens. Previous studies showed that intratumoral administration of rabbit erythrocyte-derived α-Gal glycolipids into B16 melanoma lesions led to CD8+ T cell-mediated immunity to MAAs as well as to OVA as a surrogate TSA, proving the mechanism of α-Gal glycolipid-induced anti-tumor immunity [15, 16]. As with rabbit erythrocyte-derived α-Gal glycolipids, AGI-134 causes the regression of established tumors and protects mice from the development of un-injected secondary lesions when injected into a single primary lesion, demonstrating that AGI-134 also confers anti-tumor immunity. When taken together with the in vitro cross presentation data, it can be assumed that AGI-134-induced antitumor immunity is also mediated largely by CD8+ T cells.

We have further shown that AGI-134 synergizes well with a checkpoint inhibitor. Immunotherapies that block immune checkpoints have revolutionized cancer treatment. PD-1 and cytotoxic T lymphocyte-associated protein 4 (CTLA-4) are negative regulators of T cells that promote T cell anergy in the tumor microenvironment. Monoclonal antibodies that target PD-1 and CTLA-4 promote activation and expansion of T cells by blocking these immune checkpoints and have demonstrated efficacy in a wide range of tumor types (reviewed by [59]). However, these treatments are only efficacious in a subset of patients and, particularly for anti-PD-1, activity is associated with patients who had a T cell inflamed tumor type prior to starting treatment. In addition, the side effects of autoimmune phenomena are observed in a large proportion of the patients because of activation of autoreactive T cell populations. One mechanism to boost the efficacy of anti-PD-1 antibodies is to increase the repertoire of activated tumor-specific T cells prior to treatment with anti-PD-1. Tumors have a highly diverse array of unique mutations, which result in neoantigens that can be unique between patients, lesions and even within lesions [60]. However, the immunosuppressive tumor microenvironment often means that many of these neoantigens have not been effectively processed by the immune system. As α-Gal glycolipids actively induce CD8+ T cell-mediated immunity to TSAs and overcome regulatory T cell activity [16], we hypothesized that AGI-134 would boost the efficacy of anti-PD-1 therapy. Indeed, when we treated primary B16-F10 melanoma lesions in α1,3GT^−/−^ mice with a combination of an anti-PD-1 treatment regimen that was not efficacious and a suboptimal dose of AGI-134, the degree of protection from secondary tumor development was significantly enhanced over either treatment when administered alone.

## Conclusions

In conclusion, we have identified an α-Gal glycolipid-like small molecule as an immunotherapeutic drug candidate for the treatment of solid tumors initiated by intratumoral injection. This molecule possesses the requisite properties that make it amenable for development as a human therapeutic. Anti-Gal antibodies are recruited to AGI-134 treated cells and stimulate CDC and ADCC after incubation in human serum. The CDC and ADCC-killed cells are specifically phagocytosed by APCs and associated antigen cross-presented by murine CD8+ DCs. When injected into primary melanoma lesions in mice, AGI-134 protects from the development of un-injected lesions. Finally, AGI-134 acts in synergy with an anti-PD-1 antibody, indicating that AGI-134 could be an excellent combination partner for anti-PD-1 therapy, by increasing the repertoire of tumor-specific T cells prior to anti-PD-1 treatment.

Based on the data collectively shown in this manuscript, a first-in-man clinical study with AGI-134 has been initiated in July 2018 (NCT03593226). This is a phase I/IIa, multicenter, open-label study to evaluate the safety and tolerability of escalating doses of AGI-134 given as monotherapy and in combination with pembrolizumab in unresectable metastatic solid tumors.

## Supplementary information

**Additional file 1: Figure S1.** Compound structures. (**A**) AGI-134 (functional head group is a galactose-α-1,3-galactosyl-beta-1,4-*N*-acetyl-glucosamine: α-Gal); (**B**) FSL-Fluorescein (functional group is fluorescein); (**C**) FSL-A (functional group is N-acetyl-galactosamine-α-1,3-fucosyl-α-1,2-galactose: blood group A antigen).

**Additional file 2: Figure S2.** Fc gamma (Fcγ) and complement receptor (CR) expression on differentiated human macrophages ((**A**) and (**B**), respectively). Human peripheral blood mononuclear cells were differentiated into macrophages using M-CSF. Expression of the Fcγ receptors I–III (CD16, CD32, CD64) and the complement receptors CR1 (CD35), C3R (CD11b) and C5aR (CD88) in differentiated macrophages was verified by flow cytometry with fluorescently labeled surface marker specific antibodies (grey curves) and negative control isotype antibodies (open curves) according to standard procedures.

**Additional file 3: Figure S3.** Immunocytochemistry, complement deposition, and phagocytosis control experiments. (**A**) Anti-Gal binding to AGI-134 labeled B16-F10 cells. 1 × 10^6^ B16-F10 cells were incubated with 0 or 0.5 mg AGI-134 in PBS. Subsequently, the cells were incubated with a monoclonal anti-Gal antibody. Anti-Gal binding was visualized by fluorescence microscopy with FITC-labeled secondary antibody; cell nuclei were stained with DAPI. (**B**) A549 cells were labeled with the indicated amounts of AGI-134 and then incubated with 0 or 50% normal human serum (NHS). Deposition of the complement components C3b and C5b-C9 (MAC) was detected with fluorescein-labeled anti-C3b or anti-MAC antibodies. FL-1 histogram overlays for a representative experiment of several performed are shown. (**C**) In phagocytosis experiments with the phagocytosis inhibitor cytochalasin D, Far Red CellTrace labeled human macrophages and CFSE-labeled A549 target cells were incubated with AGI-134 and NHS in the presence or absence of 5 μM cytochalasin D. Then, the cells were treated with trypsin/EDTA to dissociate A549 from the macrophages. The samples were analyzed by flow cytometry as above. Double positive events in the samples treated with trypsin/EDTA. Data shown is representative of two independent experiments: mean + SD of duplicate samples. (**D**) A549 and SW480 cells were stained with CD59, CD55 or control antibodies and analyzed by flow cytometry. Mean fold increase data shown is representative of two independent experiments: mean + SD.

**Additional file 4: Figure S4.** Binding of mouse anti-Gal antibodies and complement deposition on AGI-134-treated mouse melanoma cells. (**A**–**D**) B16-F10 or JB/RH cells were treated with the indicated AGI-134 concentrations or the negative control glycolipid FSL-A. The cells were then incubated with mouse anti-Gal IgM, serum from non-immunized (low anti-Gal titers) or PKH-immunized (high anti-Gal titers) α1,3GT^−/−^ mice, or anti-blood group A,B primary antibodies or buffer only. Anti-Gal antibody binding or deposition of the complement factors C3b/i and C5b-9 were detected using antibodies and flow cytometry. Histogram overlays for the various samples are plotted for representative data from several experiments performed. Of note, the samples for anti-FSL-A and complement deposition in (**D**) were run in parallel in the same experiment.

**Additional file 5: Figure S5.** (**A**) Anti-Gal IgG and IgM titer in PKH-immunized vs. non-immunized α1,3GT^−/−^ mice. Five α1,3GT^−/−^ mice were immunized five times with PKH. Heparinized blood before the first and after the last immunization was collected for each animal and plasma prepared. The anti-Gal IgG and IgM titers for pre-immune and post-immunization plasma were determined by ELISA with immobilized α-Gal (see methods section). The absorbance values at 492 nm (A_492_) as read-out for anti-Gal binding are plotted relative to the plasma dilutions. (**B**) Representative data from experiments where the dose-dependency of the abscopal effect of AGI-134 in B16-F10 tumors in α1,3GT^−/−^ mice was monitored over 25 days are shown. (**C**) Abscopal effect of activity of four doses of intraperitoneally (i.p.) injected anti-PD-1 antibody RMP1-14 in B16-F10 model as compared to mock treatment (Treatment start: Day 5). (**B**, **C**) Differences in secondary tumor development over time between the treatment groups were calculated by Mantel-Cox test (*, *p *< 0.05; ***, *p *< 0.0005).

## Data Availability

The datasets and materials used for the current study are available from the corresponding author on reasonable request with permission by Agalimmune Ltd., a subsidiary of BioLineRx.

## Abbreviations

7-AAD: 7-aminoactinomycin D
ADCC: antibody-dependent cellular cytotoxicity
α-Gal: galactose-α-1,3-galactosyl-beta-1,4-*N*-acetyl-glucosamine
anti-Gal: anti-α-Gal antibody
APC: antigen-presenting cell
BSA: bovine serum albumin
CDC: complement-dependent cytotoxicity
CDS: cell dissociation solution
CFSE: carboxyfluorescein succinimidyl ester
CHO-OVA: ovalbumin expressing CHO-K1 cells
CTLA-4: cytotoxic T-lymphocyte-associated protein 4
CytD: cytochalasin D
DAPI: 4′,6-diamidino-2-phenylindole
DC: dendritic cell
EtOH: ethanol
FBS: fetal bovine serum
FcγRIIIa: Fc gamma receptor 3a
FITC: fluorescein isothiocyanate
FSL: Kode Biotech function-spacer-lipid construct
GFP: green fluorescent protein
gMFI: geometric mean fluorescence intensity
GT: α-1,3-galactosyl transferase
GT KO or α1,3GT^−/−^ mice: α-1,3-galactosyl transferase knock out mice
HSA: human serum albumin
IACUC: Institutional Animal Care and Use Committee
i.p.: intraperitoneal
i.t.: intratumoral
iNHS: heat-inactivated normal human serum
MAA: melanoma associated antigen
MAC: membrane attack complex
MDM: monocyte-derived macrophages
MutuDC: murine tumor dendritic cells
NHS: normal human serum
NHSBT: National Health Service Blood and Transplant
OT-1: OVA-T cell receptor-1
OVA: ovalbumin
PBS: phosphate buffered saline
PD-1: programmed cell death 1 receptor
PDL-1: programmed death-ligand
PKH: pig kidney homogenate
1°: primary
Rag1: recombination activating gene 1
RLU: relative light units
RRBC: rabbit red blood cell
RLU: relative light units
2°: secondary
s.c.: subcutaneous
TSA: tumor-specific antigen
