# Supplementary material for: AGI-134: a fully synthetic α-Gal glycolipid that converts tumors into in situ autologous vaccines, induces anti-tumor immunity and is synergistic with an anti-PD-1 antibody in mouse melanoma models
Source: Cancer Cell Int. 2019 Dec 19;19:346. doi: 10.1186/s12935-019-1059-8 (PMC6923872; doi:10.1186/s12935-019-1059-8)
Supplement: Supplementary file 1 — Additional file 1: Figure S1. Compound structures. (A) AGI-134 (functional head group is a galactose-α-1,3-galactosyl-beta-1,4-N-acetyl-glucosamine: α-Gal); (B) FSL-Fluorescein (functional group is fluorescein); (C) FSL-A (functional group is N-acetyl-galactosamine-α-1,3-fucosyl-α-1,2-galactose: blood group A antigen). [file 12935_2019_1059_MOESM1_ESM.pptx]

## Slide 1
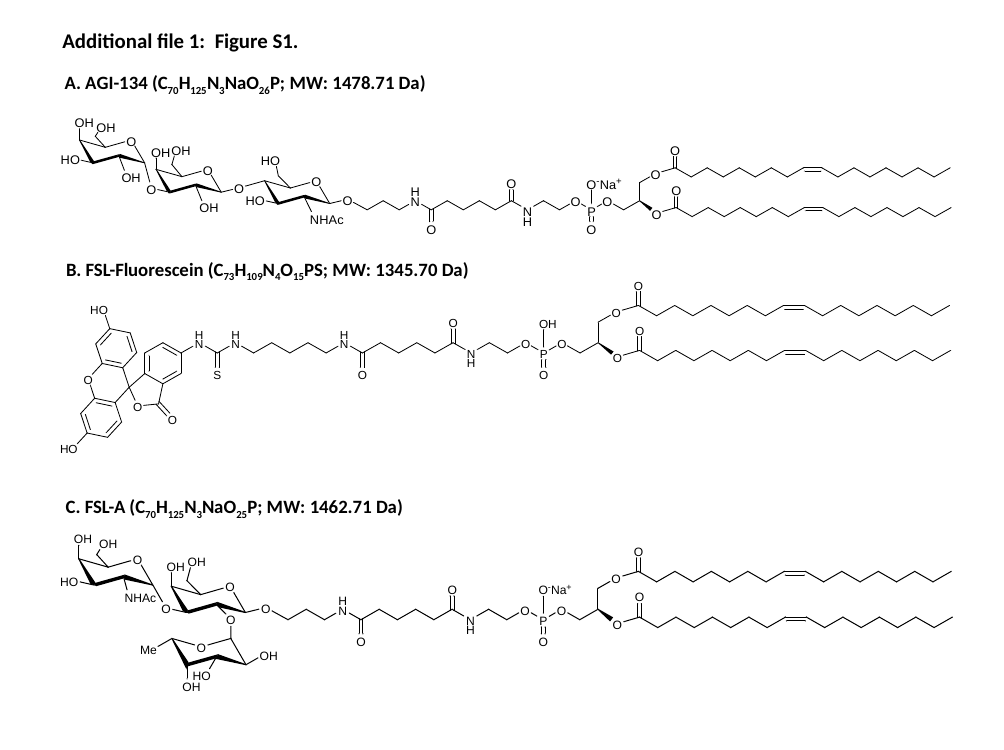

Additional file 1: Figure S1.
A. AGI-134 (C70H125N3NaO26P; MW: 1478.71 Da)
B. FSL-Fluorescein (C73H109N4O15PS; MW: 1345.70 Da)
C. FSL-A (C70H125N3NaO25P; MW: 1462.71 Da)
