# Supplementary material for: AGI-134: a fully synthetic α-Gal glycolipid that converts tumors into in situ autologous vaccines, induces anti-tumor immunity and is synergistic with an anti-PD-1 antibody in mouse melanoma models
Source: Cancer Cell Int. 2019 Dec 19;19:346. doi: 10.1186/s12935-019-1059-8 (PMC6923872; doi:10.1186/s12935-019-1059-8)
Supplement: Supplementary file 2 — Additional file 2: Figure S2. Fc gamma (Fcγ) and complement receptor (CR) expression on differentiated human macrophages ((A) and (B), respectively). Human peripheral blood mononuclear cells were differentiated into macrophages using M-CSF. Expression of the Fcγ receptors I–III (CD16, CD32, CD64) and the complement receptors CR1 (CD35), C3R (CD11b) and C5aR (CD88) in differentiated macrophages was verified by flow cytometry with fluorescently labeled surface marker specific antibodies (grey curves) and negative control isotype antibodies (open curves) according to standard procedures. [file 12935_2019_1059_MOESM2_ESM.pptx]

## Slide 1
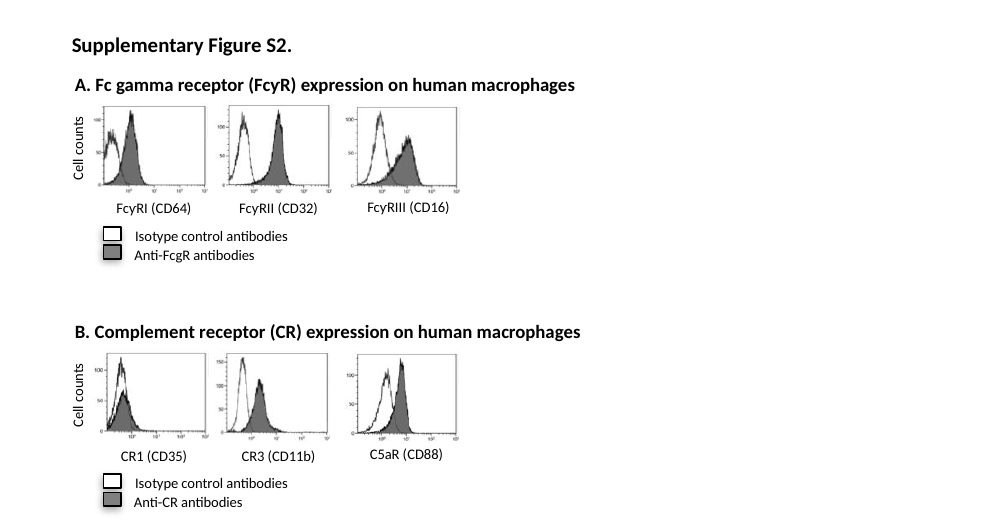

Supplementary Figure S2.
A. Fc gamma receptor (FcƴR) expression on human macrophages
Cell counts
FcƴRIII (CD16)
FcƴRII (CD32)
FcƴRI (CD64)
Isotype control antibodies
Anti-FcgR antibodies
B. Complement receptor (CR) expression on human macrophages
Cell counts
C5aR (CD88)
CR1 (CD35)
CR3 (CD11b)
Isotype control antibodies
Anti-CR antibodies
