# Supplementary material for: AGI-134: a fully synthetic α-Gal glycolipid that converts tumors into in situ autologous vaccines, induces anti-tumor immunity and is synergistic with an anti-PD-1 antibody in mouse melanoma models
Source: Cancer Cell Int. 2019 Dec 19;19:346. doi: 10.1186/s12935-019-1059-8 (PMC6923872; doi:10.1186/s12935-019-1059-8)
Supplement: Supplementary file 3 — Additional file 3: Figure S3. Immunocytochemistry, complement deposition, and phagocytosis control experiments. (A) Anti-Gal binding to AGI-134 labeled B16-F10 cells. 1 × 10^6^ B16-F10 cells were incubated with 0 or 0.5 mg AGI-134 in PBS. Subsequently, the cells were incubated with a monoclonal anti-Gal antibody. Anti-Gal binding was visualized by fluorescence microscopy with FITC-labeled secondary antibody; cell nuclei were stained with DAPI. (B) A549 cells were labeled with the indicated amounts of AGI-134 and then incubated with 0 or 50% normal human serum (NHS). Deposition of the complement components C3b and C5b-C9 (MAC) was detected with fluorescein-labeled anti-C3b or anti-MAC antibodies. FL-1 histogram overlays for a representative experiment of several performed are shown. (C) In phagocytosis experiments with the phagocytosis inhibitor cytochalasin D, Far Red CellTrace labeled human macrophages and CFSE-labeled A549 target cells were incubated with AGI-134 and NHS in the presence or absence of 5 μM cytochalasin D. Then, the cells were treated with trypsin/EDTA to dissociate A549 from the macrophages. The samples were analyzed by flow cytometry as above. Double positive events in the samples treated with trypsin/EDTA. Data shown is representative of two independent experiments: mean + SD of duplicate samples. (D) A549 and SW480 cells were stained with CD59, CD55 or control antibodies and analyzed by flow cytometry. Mean fold increase data shown is representative of two independent experiments: mean + SD. [file 12935_2019_1059_MOESM3_ESM.pptx]

## Slide 1
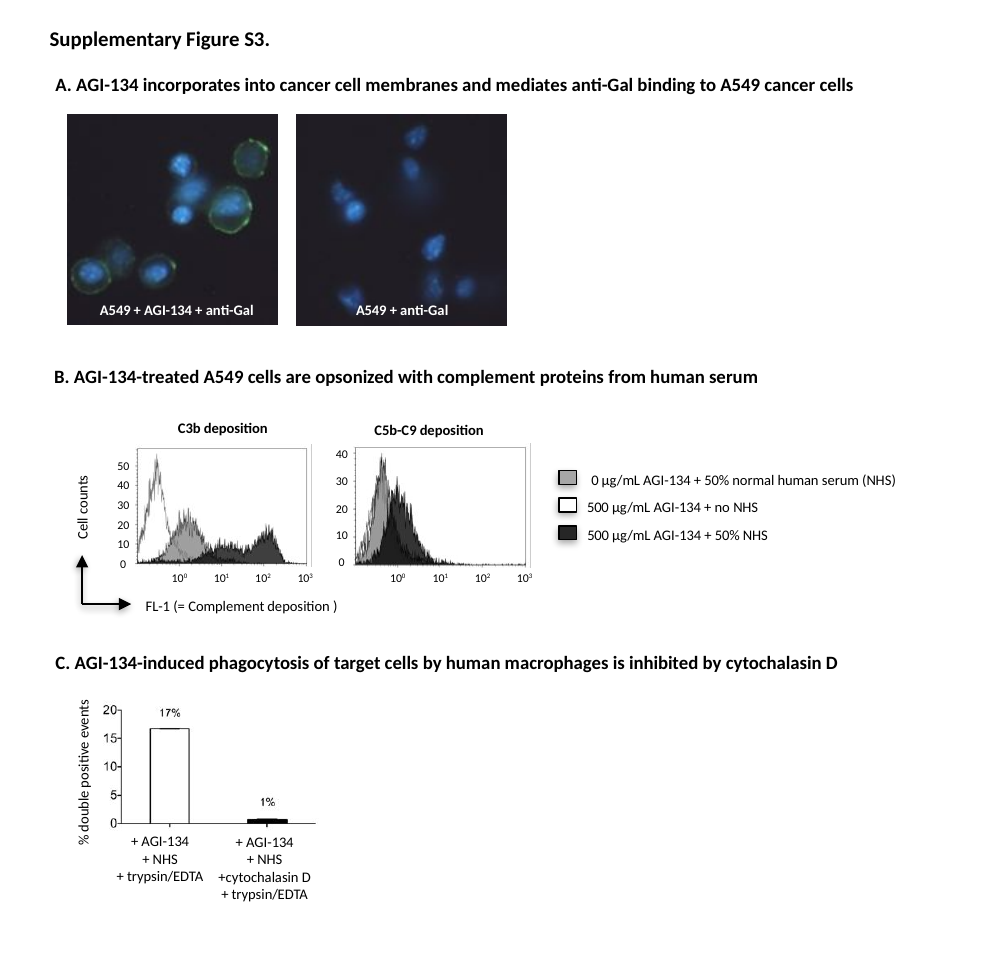

Supplementary Figure S3.
A. AGI-134 incorporates into cancer cell membranes and mediates anti-Gal binding to A549 cancer cells
A549 + AGI-134 + anti-Gal
A549 + anti-Gal
B. AGI-134-treated A549 cells are opsonized with complement proteins from human serum
C3b deposition
C5b-C9 deposition
40
50
Cell counts
FL-1 (= Complement deposition )
 0 µg/mL AGI-134 + 50% normal human serum (NHS)
500 µg/mL AGI-134 + no NHS
500 µg/mL AGI-134 + 50% NHS
30
40
30
20
20
10
10
0
0
101
102
103
100
101
102
103
100
C. AGI-134-induced phagocytosis of target cells by human macrophages is inhibited by cytochalasin D
% double positive events
+ AGI-134
+ NHS
+ trypsin/EDTA
+ AGI-134
+ NHS
+cytochalasin D
+ trypsin/EDTA
