# Supplementary material for: AGI-134: a fully synthetic α-Gal glycolipid that converts tumors into in situ autologous vaccines, induces anti-tumor immunity and is synergistic with an anti-PD-1 antibody in mouse melanoma models
Source: Cancer Cell Int. 2019 Dec 19;19:346. doi: 10.1186/s12935-019-1059-8 (PMC6923872; doi:10.1186/s12935-019-1059-8)
Supplement: Supplementary file 4 — Additional file 4: Figure S4. Binding of mouse anti-Gal antibodies and complement deposition on AGI-134-treated mouse melanoma cells. (A–D) B16-F10 or JB/RH cells were treated with the indicated AGI-134 concentrations or the negative control glycolipid FSL-A. The cells were then incubated with mouse anti-Gal IgM, serum from non-immunized (low anti-Gal titers) or PKH-immunized (high anti-Gal titers) α1,3GT−/− mice, or anti-blood group A,B primary antibodies or buffer only. Anti-Gal antibody binding or deposition of the complement factors C3b/i and C5b-9 were detected using antibodies and flow cytometry. Histogram overlays for the various samples are plotted for representative data from several experiments performed. Of note, the samples for anti-FSL-A and complement deposition in (D) were run in parallel in the same experiment. [file 12935_2019_1059_MOESM4_ESM.pptx]

## Slide 1
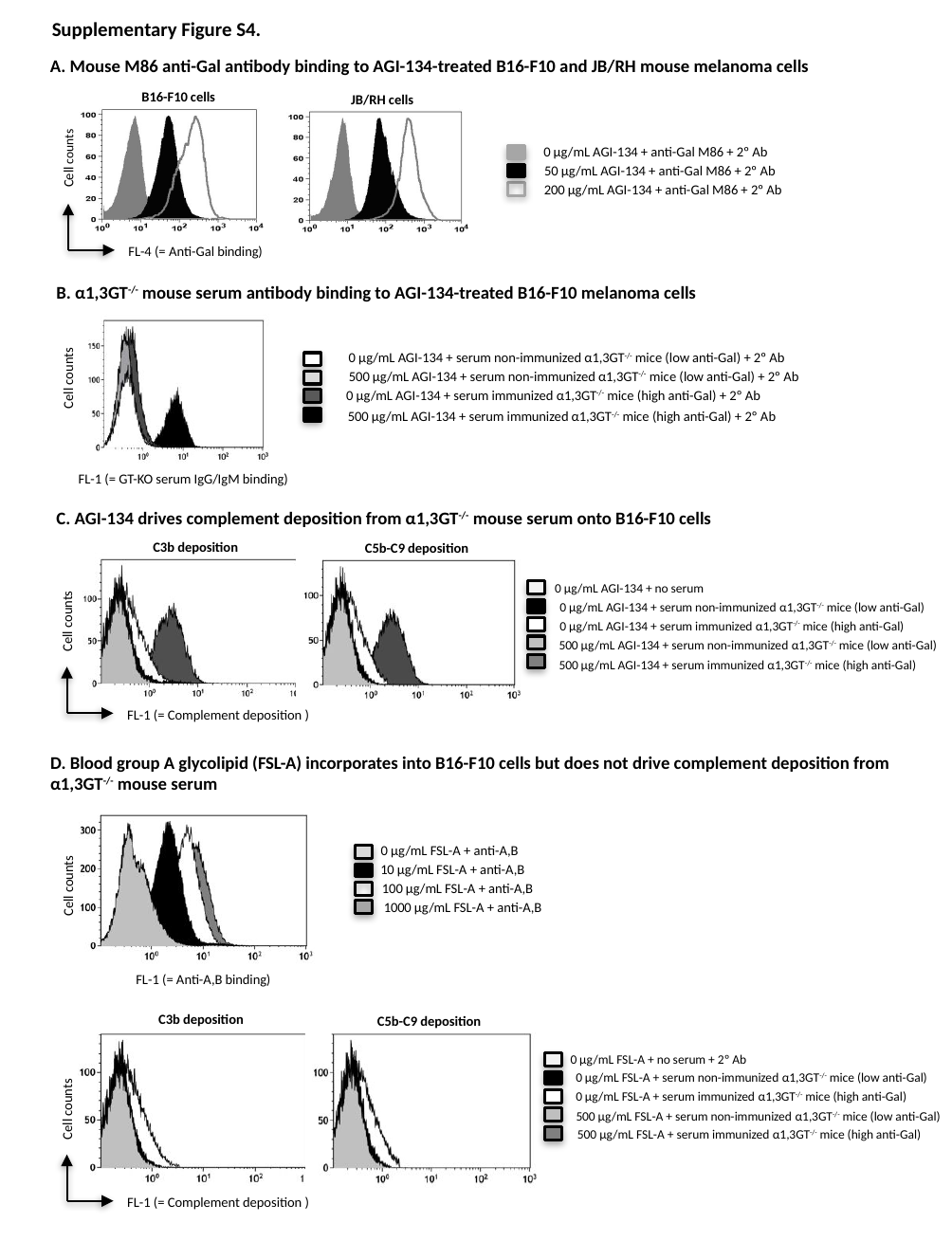

Supplementary Figure S4.
A. Mouse M86 anti-Gal antibody binding to AGI-134-treated B16-F10 and JB/RH mouse melanoma cells
B16-F10 cells
JB/RH cells
Cell counts
FL-4 (= Anti-Gal binding)
 0 µg/mL AGI-134 + anti-Gal M86 + 2º Ab
 50 µg/mL AGI-134 + anti-Gal M86 + 2º Ab
200 µg/mL AGI-134 + anti-Gal M86 + 2º Ab
B. α1,3GT-/- mouse serum antibody binding to AGI-134-treated B16-F10 melanoma cells
 0 µg/mL AGI-134 + serum non-immunized α1,3GT-/- mice (low anti-Gal) + 2º Ab
 500 µg/mL AGI-134 + serum non-immunized α1,3GT-/- mice (low anti-Gal) + 2º Ab
 0 µg/mL AGI-134 + serum immunized α1,3GT-/- mice (high anti-Gal) + 2º Ab
 500 µg/mL AGI-134 + serum immunized α1,3GT-/- mice (high anti-Gal) + 2º Ab
Cell counts
FL-1 (= GT-KO serum IgG/IgM binding)
C. AGI-134 drives complement deposition from α1,3GT-/- mouse serum onto B16-F10 cells
C3b deposition
C5b-C9 deposition
 0 µg/mL AGI-134 + no serum
Cell counts
FL-1 (= Complement deposition )
 0 µg/mL AGI-134 + serum non-immunized α1,3GT-/- mice (low anti-Gal)
 0 µg/mL AGI-134 + serum immunized α1,3GT-/- mice (high anti-Gal)
500 µg/mL AGI-134 + serum non-immunized α1,3GT-/- mice (low anti-Gal)
500 µg/mL AGI-134 + serum immunized α1,3GT-/- mice (high anti-Gal)
D. Blood group A glycolipid (FSL-A) incorporates into B16-F10 cells but does not drive complement deposition from α1,3GT-/- mouse serum
 0 µg/mL FSL-A + anti-A,B
 10 µg/mL FSL-A + anti-A,B
 100 µg/mL FSL-A + anti-A,B
1000 µg/mL FSL-A + anti-A,B
Cell counts
FL-1 (= Anti-A,B binding)
C3b deposition
C5b-C9 deposition
 0 µg/mL FSL-A + no serum + 2º Ab
 0 µg/mL FSL-A + serum non-immunized α1,3GT-/- mice (low anti-Gal)
 0 µg/mL FSL-A + serum immunized α1,3GT-/- mice (high anti-Gal)
Cell counts
500 µg/mL FSL-A + serum non-immunized α1,3GT-/- mice (low anti-Gal)
500 µg/mL FSL-A + serum immunized α1,3GT-/- mice (high anti-Gal)
FL-1 (= Complement deposition )
