# Supplementary material for: AGI-134: a fully synthetic α-Gal glycolipid that converts tumors into in situ autologous vaccines, induces anti-tumor immunity and is synergistic with an anti-PD-1 antibody in mouse melanoma models
Source: Cancer Cell Int. 2019 Dec 19;19:346. doi: 10.1186/s12935-019-1059-8 (PMC6923872; doi:10.1186/s12935-019-1059-8)
Supplement: Supplementary file 5 — Additional file 5: Figure S5. (A) Anti-Gal IgG and IgM titer in PKH-immunized vs. non-immunized α1,3GT−/− mice. Five α1,3GT−/− mice were immunized five times with PKH. Heparinized blood before the first and after the last immunization was collected for each animal and plasma prepared. The anti-Gal IgG and IgM titers for pre-immune and post-immunization plasma were determined by ELISA with immobilized α-Gal (see methods section). The absorbance values at 492 nm (A492) as read-out for anti-Gal binding are plotted relative to the plasma dilutions. (B) Representative data from experiments where the dose-dependency of the abscopal effect of AGI-134 in B16-F10 tumors in α1,3GT−/− mice was monitored over 25 days are shown. (C) Abscopal effect of activity of four doses of intraperitoneally (i.p.) injected anti-PD-1 antibody RMP1-14 in B16-F10 model as compared to mock treatment (Treatment start: Day 5). (B, C) Differences in secondary tumor development over time between the treatment groups were calculated by Mantel-Cox test (*, p < 0.05; ***, p < 0.0005). [file 12935_2019_1059_MOESM5_ESM.pptx]

## Slide 1
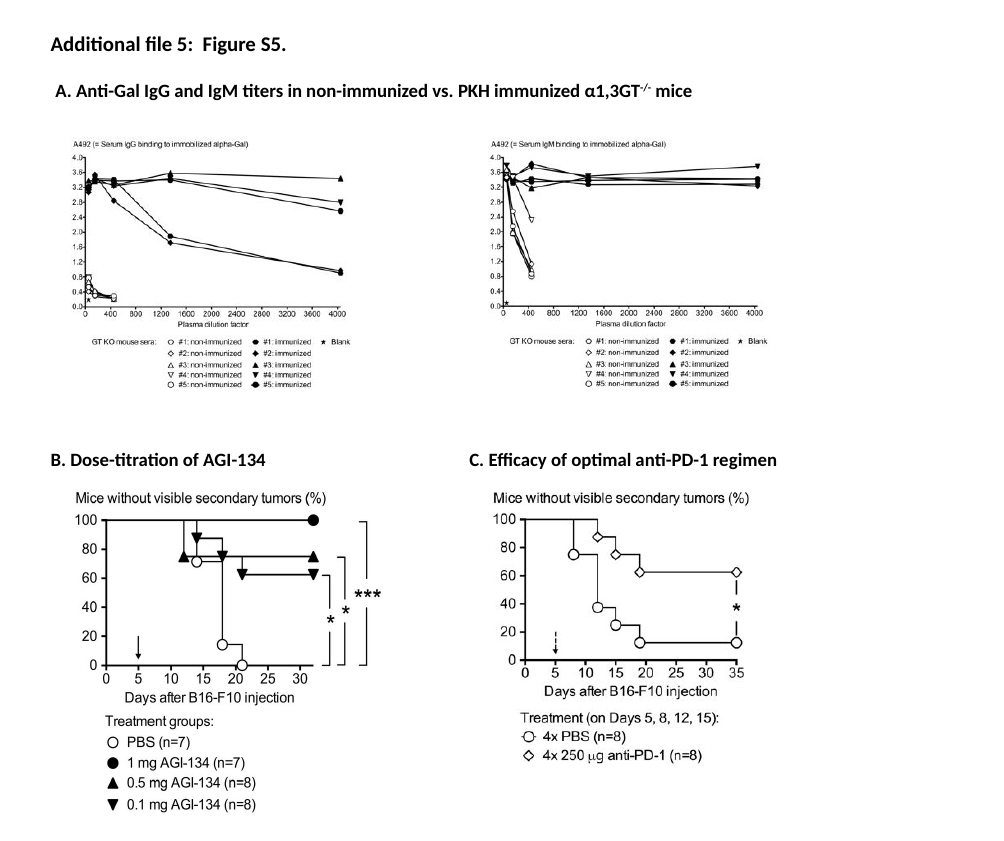

Additional file 5: Figure S5.
A. Anti-Gal IgG and IgM titers in non-immunized vs. PKH immunized α1,3GT-/- mice
C. Efficacy of optimal anti-PD-1 regimen
B. Dose-titration of AGI-134
